# Functional Information Stored in the Conserved Structural RNA Domains of Flavivirus Genomes

**DOI:** 10.3389/fmicb.2017.00546

**Published:** 2017-04-03

**Authors:** Alba Fernández-Sanlés, Pablo Ríos-Marco, Cristina Romero-López, Alfredo Berzal-Herranz

**Affiliations:** Department of Molecular Biology, Instituto de Parasitología y Biomedicina “López-Neyra,” Consejo Superior de Investigaciones Científicas (IPBLN-CSIC)Granada, Spain

**Keywords:** flavivirus RNA genome, functional RNA domains, RNA–RNA interactions, RNA structure/function, DENV, WNV

## Abstract

The genus *Flavivirus* comprises a large number of small, positive-sense single-stranded, RNA viruses able to replicate in the cytoplasm of certain arthropod and/or vertebrate host cells. The genus, which has some 70 member species, includes a number of emerging and re-emerging pathogens responsible for outbreaks of human disease around the world, such as the West Nile, dengue, Zika, yellow fever, Japanese encephalitis, St. Louis encephalitis, and tick-borne encephalitis viruses. Like other RNA viruses, flaviviruses have a compact RNA genome that efficiently stores all the information required for the completion of the infectious cycle. The efficiency of this storage system is attributable to supracoding elements, i.e., discrete, structural units with essential functions. This information storage system overlaps and complements the protein coding sequence and is highly conserved across the genus. It therefore offers interesting potential targets for novel therapeutic strategies. This review summarizes our knowledge of the features of flavivirus genome functional RNA domains. It also provides a brief overview of the main achievements reported in the design of antiviral nucleic acid-based drugs targeting functional genomic RNA elements.

## Introduction

The great plasticity of RNA virus genomes allows them to perform different functions during the infectious cycle, helping viral populations adapt to novel molecular and cellular contexts, and to escape host defenses. It also contributes toward the development of resistance to antiviral drugs. These feats are achieved by the genome preserving a degree of variability while avoiding challenges to viral fitness. Genome variability can become a threat to viral survival if it reaches the error catastrophe limit (Schuster, 1993; Eigen, 2002), but RNA viruses have overcome this by storing information required for essential functions in discrete, highly conserved, genomic RNA structural domains. These complexly folded regions may overlap the nucleotide sequence coding for viral proteins. They play out their different biological roles (e.g., in replication, translation, or encapsidation) by directly recruiting viral and/or cellular factors, or by forming high-order regulatory structures via the establishment of long-range RNA–RNA interaction networks resulting in the formation of the complex global structures required for correct viral functioning. By means of this dynamic folding, the RNA genome can perform functions during the viral cycle other than simply coding for proteins (Romero-López and Berzal-Herranz, 2013).

*Flavivirus* spp. (from now on flaviviruses) belong to the family *Flaviviridae*. They are small (40–65 nm diameter), enveloped (icosahedral nucleocapsid) viruses with a positive single-stranded RNA genome. The genus includes important human pathogens responsible for ongoing/recurrent outbreaks of disease in areas where such diseases are not traditionally endemic; West Nile virus (WNV), dengue virus (DENV, perhaps the most important human pathogen of the genus) and Zika virus (ZIKV), for example, are all dramatically expanding their original geographic distribution. Other well-known flaviviruses include the causal agents of yellow fever (YFV), Japanese encephalitis (JEV), St. Louis encephalitis (SLEV), Murray Valley encephalitis (MVEV), or tick-borne encephalitis (TBEV) among of over 70 flaviviruses that have been identified. Some authors believe there could be over 2,000 left to discover (Pybus et al., 2002).

Most flaviviruses are transmitted to vertebrate hosts by the bite of haematophagous arthropods (thus classifying them within the heterogeneous group of arboviruses). Flaviviruses have traditionally been assigned to one of three clusters according to their arthropod vectors (Kuno et al., 1998; Cook and Holmes, 2006; Cook et al., 2012): mosquito-borne (MBFV), tick-borne (TBFV), and no-known-vector (NKV) flaviviruses (**Table 1**). These clusters can be further divided into clades and species. The members of the MBFV and TBFV clusters replicate in vertebrates and arthropods, while the NKV flaviviruses can be subdivided into two clades infecting solely bats or rodents, with no arthropod vector involved in the infective cycle. A fourth cluster, gathers together the insect-specific flaviviruses (ISFV), has recently been defined and characterized (Cook et al., 2012). It is the most divergent group and can be subdivided according to the mosquito host involved (mainly *Aedes* spp. and *Culex* spp.). ISFVs do not infect any vertebrate host (**Table 1**). Finally, Tamana bat virus (TABV), which infects exclusively mammalian cells, shows no serological relationship with any other flavivirus, and has only very distant phylogenetic relationships with them. Its taxonomic position, therefore, is not well defined (de Lamballerie et al., 2002; Roby et al., 2014).

**Table 1:** Classification of flaviviruses.

Flaviviruses	Abbreviation	Primary host (^∗^)
Mosquito-borne flaviviruses	MBFV	
**Dengue virus group**	DENV group	Primates
Dengue virus serotype 1	DENV-1	
Dengue virus serotype 2	DENV-2	
Dengue virus serotype 3	DENV-3	
Dengue virus serotype 4	DENV-4	
**Japanese encephalitis virus group**	JEV group	Birds
Japanese encephalitis virus	JEV	
West Nile virus	WNV	
Murray Valley encephalitis virus	MVEV	
St. Louis encephalitis virus	SLEV	
Usutu virus	USUV	
**Spondweni virus group**	SPOV group	Primates
Zika virus	ZIKV	
**Yellow fever virus group**	YFV group	Primates
Yellow fever virus	YFV	
**Aroa virus group**	AROAV group	Not determined
**Kokobera virus group**	KOKV group	Macropods
**Ntaya virus group**	NTAV	Birds
Tick-borne flaviviruses	TBFV	
**Mammalian tick-borne virus group**		Rodents
Tick-borne encephalitis virus	TBEV	
Langat virus	LGTV	
Powassan virus	POWV	
Ngiye virus	NGOV	
**Seabird tick-borne virus group**		Seabirds
Kama virus	KAMV	
Meaban virus	MEAV	
Saumarez Reef virus	SREV	
No-known-vector flaviviruses	NKV	
**Modoc virus group**	MODV	Rodents
**Rio bravo virus group**	RBV	Bats
Insect-specific flaviviruses	ISFV	Mosquitoes
**Classical ISFVs**	cISFV	
Cell fusing agent virus	CFAV	
**Dual host affiliated ISFVs**	dISFV	
Lammi virus	LAMV	
Tamana bat virus	TABV	Bats

^∗^*Always a vertebrate host, except for ISF, which infects mosquitoes. Representative examples of different groups are included*.

Certainly, flaviviruses pose health problems for humans (and some other vertebrates) that may be associated with enormous social and economic costs. Over the last decade, the number of outbreaks of flavivirus-induced disease has increased all over the world. The main causes include the geographic expansion of their mosquito vectors, and increasing human travel to the areas of highest infection risk. They cause non-specific symptoms in the initial phase of infection in humans, which hinders their control, and as for other RNA viruses, no efficient therapeutic or immunoprophylactic strategies have been developed. The World Health Organization^1^ and the Centers for Disease Control^2^ therefore both cite flaviviruses as a global health threat.

The functional importance of the highly conserved structural genomic RNA domains in different RNA viruses (Romero-López and Berzal-Herranz, 2013) renders them potential therapeutic targets for new antiviral drugs. This review focuses on the role of the functionally active structural RNA domains identified in the flavivirus genome. Their mechanisms of action in the regulation of essential functions of the viral cycle are discussed, and a short overview is provided of the flavivirus subgenomic RNAs (sfRNAs). Recent advances in the development of novel therapeutic strategies entailing the use of nucleic acid-based agents to target RNA molecules are also described.

## The Flavivirus Infectious Cycle

### Cell Entry and Internalization

**FIGURE 1:**
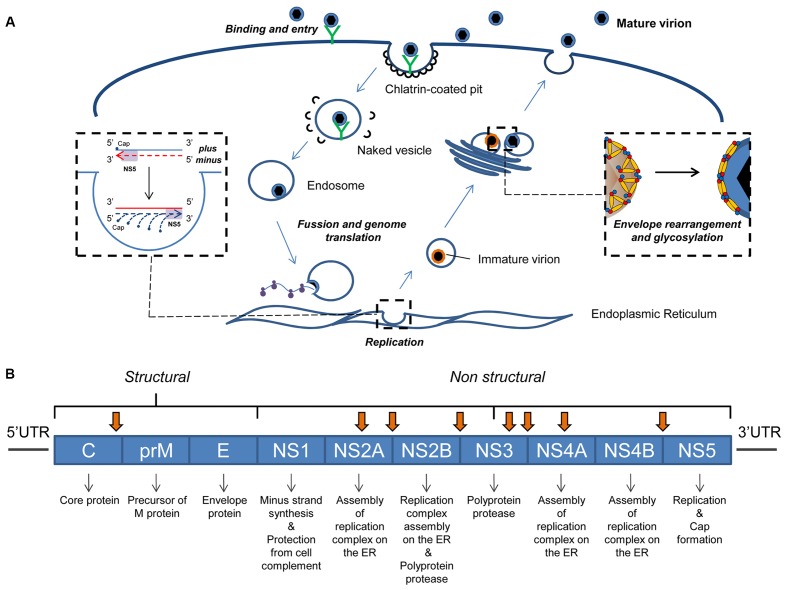
**Flavivirus infective cycle and genome. (A)** Diagram of flavivirus infective cycle. **(B)** Genetic organization of flavivirus genomes. These genomes code for three structural and seven non-structural proteins, the main functions of which are shown. Arrows indicate the cleavage sites for the viral protease NS2B/NS3 (Bera et al., 2007).

The mechanism by which flaviviral particles attach to the cell membrane is only partially understood. It has been reported that host surface glycoproteins interact with the viral envelope proteins to initiate attachment (Chen et al., 1997b; Kroschewski et al., 2003; Davis et al., 2006). Attachment might also be mediated by integrins, cytoskeleton proteins, and cholesterol-dependent lipid raft pathways (Medigeshi et al., 2008; Bogachek et al., 2010). Internalization is then mediated by clathrin-coated vesicles (Chu and Ng, 2004). The subsequent acidification of these vesicles causes the viral capsid proteins to fuse with the vesicle membrane, releasing the viral genome into the cytoplasm (Allison et al., 1995) (**Figure 1A**). This then reaches the surface of the endoplasmic reticulum (ER) where the molecular environment that allows the viral cycle to proceed is created, while preventing interferon response signaling (Hoenen et al., 2007; Welsch et al., 2009).

### Translation and Replication

During the early phase of the flavivirus cycle, the viral genome is mainly employed as mRNA in viral protein synthesis. The initiation of translation occurs in a cap-dependent manner. In DENV, the process starts with the binding of the eukaryotic initiation factor 4E (eIF4E) to the 5′cap, and the further recruitment of eIF4G and eIF4A (Merrick, 2004; Paranjape and Harris, 2010). This ribonucleoprotein complex binds to the 43S particle (40S + eIF1A + eIF3) and the AUG start codon can then be recognized (Khromykh and Westaway, 1997). Finally, the 60S ribosomal subunit is recruited and translation starts. The resulting polyprotein product is cleaved by viral and host proteases into three structural (capsid C, precursor of membrane prM, and envelope E) and seven non-structural (NS1, NS2A, NS2B, NS3, NS4A, NS4B, and NS5) proteins (Nowak et al., 1989) (**Figure 1B**).

Once viral proteins levels are appropriate, the ER membrane undergoes structural rearrangements that promote the formation of replication complexes (Welsch et al., 2009; Gillespie et al., 2010; Kaufusi et al., 2014). In addition to the circular RNA genome conformation – stabilized by long-distance 3′–5′ interactions (see below) (Khromykh et al., 2001; Zhang et al., 2008a) – and viral proteins, different host cell factors including AUF1 (Friedrich et al., 2014), eEF1A (p52) (Brinton, 2001), the TIAR (T-cell intracellular antigen-related), and TIA-1 (T-cell intracellular antigen-1) proteins (Li et al., 2002; Emara and Brinton, 2007), La protein (Vashist et al., 2009), PABP (Polacek et al., 2009b) and PTB (polypyrimidine tract binding protein) (Agis-Juarez et al., 2009; Anwar et al., 2009) are thought necessary for the completion of the viral cycle.

It has been recently shown that flaviviruses suppress host protein synthesis in human cells early post infection (host translation shutoff) while viral RNA translation is maintained (Roth et al., 2017). This strategy to ensure an efficient viral cycle consecution has been widely reported for other arboviruses such as the alphaviruses. In the case of flaviviruses, the precise molecular mechanisms leading to the translation shutoff remains elusive. It does not respond to the canonical pathways of translation control; several and not exclusive mechanisms might be involved in the host translation suppression (Roth et al., 2017). It is worth noting that this process is coupled to a switch from cap-dependent to cap-independent viral protein synthesis (Edgil et al., 2006; Roth et al., 2017). By a non-IRES mediated mechanism, flavivirus genome can subvert the lack of eIF4E to initiate viral translation in a 5′ and 3′ UTR dependent manner. Under these conditions, both ends of the viral genome are brought together to initiate the direct recruitment of translation initiation factors, thus by-passing the eIF4E requirements. This fact confers to the viral genome the great advantage of being able to translate viral proteins under limiting protein synthesis conditions, as highly differentiated cells (Edgil et al., 2006).

### Assembly of Structural Proteins for Virion Formation

Newly synthesized viral RNA genomes are assembled with structural proteins to form new, infectious particles. The genome packaging process is guided by mature viral capsid protein (C) at the ER (Schrauf et al., 2009). The resulting nucleocapsid is enveloped by a lipid bilayer belonging to the host cell (Chu and Ng, 2004) with the prM and E proteins embedded in it. These immature virions are transported to the Golgi, where the E and prM proteins are modified to yield the mature virion. The acidic pH of the Golgi causes a conformational rearrangement in which immature viruses lose their spiky prM-E trimer projections and acquire a smooth surface composed of E homodimers (Mukhopadhyay et al., 2005) (**Figure 1A**). Finally, the infectious particles are released by exocytosis at 8–10 h post infection (hpi). Peak extracellular virus titres are usually observed at 18–24 hpi (Chu and Ng, 2004).

## The Flavivirus Genome

The flaviviral genome consists on a positive-sense single-stranded RNA molecule approximately 11,000 nt long, varying depending on the species. It bears a type 1 cap structure at its 5′ end (m^7^GpppAmp) (Ray et al., 2006; Zhou et al., 2007; Saeedi and Geiss, 2013) but it lacks a polyA tail in the 3′ end (Wengler, 1981; Brinton et al., 1986). The RNA genome contains a single ORF flanked by untranslated regions (UTRs) (Castle et al., 1985; Wengler et al., 1985; Castle and Wengler, 1987). It serves as a messenger for the synthesis of a single polyprotein that is processed by viral and cellular proteases (Brinton, 2002) to yield 10 different products (Rice et al., 1985) (**Figure 1B**). The flanking UTRs are defined by discrete, functionally active structural RNA elements that play important roles in the viral cycle. These can be divided into essential partners in the infection process (e.g., promoters) and other elements not essential for viral RNA propagation but which help to regulate the processes involved. The functional RNA elements of all flaviviruses appear as highly conserved, complex folding regions, despite the lack of extensive sequence conservation across the *Flavivirus* genus (Brinton, 2014).

### The 5′ End of the Genomic RNA

**FIGURE 2:**
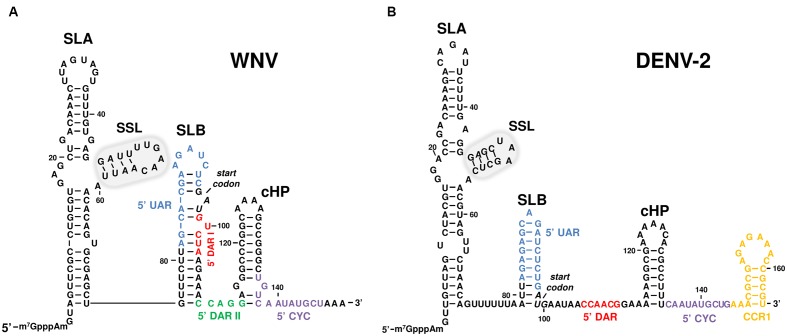
**Sequence and secondary structure of the 5′UTR of representative flavivirus genomes**. The figure shows the 5′ end of two representative MBFV genomes, **(A)** West Nile (WNV) and **(B)** dengue virus serotype 2 (DENV-2), including the functional RNA domains SLA, SSL (encircled by a gray line) SLB and cHP. The translation start codon is indicated in italics. Sequence motifs involved in viral genome cyclization are shown in colors: 5′UAR in blue, 5′DARI in red, 5′DARII in green, 5′CYC in purple, and CCR1 in orange. Sequence numbering corresponds to **(A)** the Kunjin virus MRM 61C strain (GenBank accession number L24511.1) and **(B)** DENV-2 16681 strain (GenBank accession number NC_001474).

Various functional RNA elements have been identified in the 100 nt-long 5′UTR and the 5′ end of the coding sequence of the flavivirus genome (Brinton and Dispoto, 1988; Liu et al., 2013) (**Figure 2**). The 5′UTR is relatively short in comparison with that of the IRES-dependent members of the *Flaviviridae* family. Different isolates of the same flavivirus show strong sequence conservation, and significant identity is observed among members of the same flavivirus group. Less nucleotide conservation is seen among members of different groups (Brinton and Dispoto, 1988), in contrast with the observed conservation in RNA folding (Cahour et al., 1995; Thurner et al., 2004). Preliminary structural studies of this region suggested the predicted secondary structures to be functionally important – due to their similar size and shape – in different flavivirus genomes (Brinton and Dispoto, 1988; Gritsun et al., 1997; Leyssen et al., 2002; Gritsun and Gould, 2007b). Further studies support the requirement of these functional structural elements for RNA synthesis both *in vitro* and in cell culture (Cahour et al., 1995; Filomatori et al., 2006; Lodeiro et al., 2009; Li et al., 2010). The functional role of the 5′UTR elements in RNA replication and translation has been examined mostly in DENV and extrapolated to other flavivirus (Cahour et al., 1995; Filomatori et al., 2006; Lodeiro et al., 2009). Here we focus on MBFV flavivirus genome 5′ structures (**Figure 2**).

The ∼70 nt-long domain at the extreme 5′ terminus is known as the SLA element, and this is conserved across all flavivirus groups. It folds into a Y-shape and has a main stem-loop structural element plus a smaller side stem-loop (SSL) that emerges from it. The size of the essential SSL stem and the sequence of its loop vary across flaviviruses (Leyssen et al., 2002; Thurner et al., 2004; Filomatori et al., 2006; Gritsun and Gould, 2007b; Dong et al., 2008) (**Figure 2**). The SLA architecture is recognized by viral RNA polymerase (NS5), an RNA-dependent-RNA polymerase (RdRp) involved in viral replication (Filomatori et al., 2006). It has been reported that residues located at the basal portion of the stem-loop, in the upper stem, and in the internal loop, are critical for NS5 binding and activity (Dong et al., 2008; Li et al., 2010). In addition, the SLA element is involved in directing the addition of the cap structure at the 5′ end of the viral genome during RNA synthesis (Zhou et al., 2007; Zhang et al., 2008c). This is catalyzed by the guanylyl- and methyltransferase (MTase) activities of NS5 RNA-dependent-RNA polymerase (RdRp), and requires the relocation of the 5′ end of the nascent genomic transcript at the MTase active site (Ray et al., 2006). This event seems to be dependent on the local conformation of the RNA. These features make the SLA element an essential partner in viral translation and replication (Ray et al., 2006; Li et al., 2010). This observation is reinforced by the fact that the folding of SLA is preserved across flaviviruses, regardless of any sequence differences in this region (Filomatori et al., 2006; Lodeiro et al., 2009).

In most flaviviruses, including DENV and WNV, a second, smaller stem-loop (SLB) is present downstream of SLA that shows a certain variability in its size and shape (Brinton and Dispoto, 1988). The SLB element bears the AUG translation initiation codon, which is embedded within its stem portion in a poor Kozak initiation context in many MBFVs, but in a strong context in TBFVs (Clyde and Harris, 2006; Clyde et al., 2008) (**Figure 2**). An oligo(U) tract providing an at-least-10 nt spacer between the two stem-loop structures has been observed in DENV (Lodeiro et al., 2009). In WNV, two sequence stretches – UAR and DAR I – involved in genome cyclization are embedded within this structural domain. DENV, however, contains only UAR (**Figure 2**, see also genome cyclization section).

The stable, highly conserved hairpin cHP follows the SLB element at its 3′ end, and expands into the first nucleotides of the capsid coding region of DENV and WNV (**Figure 2**). It was first identified in DENV, and despite the reduced conservation of its sequence it was predicted to be preserved in the mosquito- and TBFV flaviviruses (Clyde and Harris, 2006). cHP governs the selection of the translation initiation codon by directly positioning the ribosomal complex close to the “functional” AUG in the SLB element. Importantly, translation initiation efficiency at the appropriate codon is related to the thermodynamic stability of the cHP element (Clyde and Harris, 2006). It is reported that the introduction of stable secondary structural elements (e.g., stem-loops) downstream of an AUG codon embedded in a poor Kozak context, improves the recognition of the optimal starting triplet by pausing the translation machinery, which must unwind the hairpin (Kozak, 1990). This favors prolonged contact with the correct AUG start codon. Thus, cHP acts as a translation enhancer. In addition, it has been shown to have a role as a *cis*-replicating element in WNV and DENV (Clyde et al., 2008). cHP thus became the first known functional RNA domain with a dual functional role in the flaviviruses life cycle (Clyde et al., 2008), highlighting the efficiency of the information coding system based on structural RNA units. During early infection, translation initiation is promoted. At this stage, the viral genome has not acquired the replication competent circular conformation, but rather exhibits an extended cHP stem-loop which temporarily makes the ribosomal complex linger at the correct AUG start codon to favor its recognition. The switch to replication might occur through the establishment of long-distance RNA–RNA interactions between the 5′ and 3′ genome ends (see below). These contacts induce the acquisition of a circular conformation, which determines a slight shortening of the cHP stem, thus allowing for rearrangements in the translational-competent scaffold and the further recruitment of factors required for viral RNA synthesis (Clyde et al., 2008).

Another conserved domain within the capsid coding region – the conserved capsid-coding region 1 (CCR1; **Figure 2B**) – was first described in the DENV genome. It was shown to modulate the DENV life cycle in mammalian and mosquito cells, likely acting during a post-RNA synthesis stage and possibly regulating viral assembly (Groat-Carmona et al., 2012). It was later found in TBEV, in which it was shown to be important for efficient viral translation (Rouha et al., 2011). Despite its high sequence and structure conservation in DENV and TBEV serogroups, it is not well-conserved across the flavivirus genus (Groat-Carmona et al., 2012).

### The 3′ End of the Genomic RNA

**FIGURE 3:**
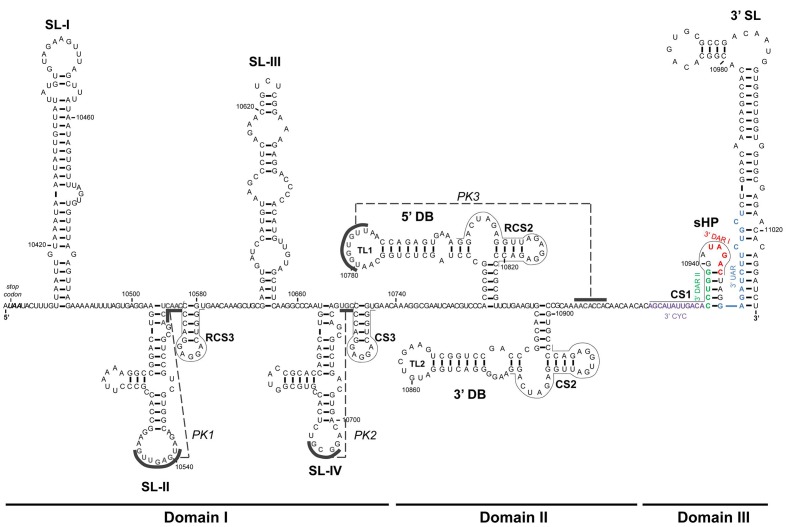
**Sequence and secondary structure of the 3′UTR**. The figure shows the 3′UTR of the WNV RNA genome. The 3′UTR is organized into three domains – I, II, and III – composed of well-defined structural elements the names of which are indicated in bold. The UAA stop codon is shown in italics. Conserved sequence motifs (CS and RCS) are indicated by thin lines. The color code for cyclization sequences is as in **Figure 2**. The pseudoknot elements (PK1, PK2, and PK3) are indicated by dashed lines, and the corresponding interacting sequences by solid lines. Sequence numbering corresponds to the Kunjin virus MRM 61C strain (GenBank accession number L24512.1).

The 3′ end of the genome terminates in a 700 nt-long untranslated region (3′UTR) that lacks a poly(A) tail. It ends in a conserved CU_OH_ dinucleotide (Wengler, 1981; Brinton and Dispoto, 1988) in MBFV and TBFV, except in some strains of TBEV (Mandl et al., 1991). The 3′UTR of flavivirus genomes is essential for viral replication (Men et al., 1996; Zeng et al., 1998). Its structure and functional characterization has mostly been deciphered in MBFVs. The 3′UTR can be subdivided into three autonomously folded regions, domains I-III (**Figure 3**), which show different degrees of sequence and structure conservation across members of the genus, with the 3′ extreme region – known as small hairpin 3′ stem-loop (sHP-3′SL) – the most conserved of all. A defining feature within the 3′UTR is the presence of duplications of structural cassettes. These are composed of various structural elements in MBFV and TBFV, but not in ISFV or NKV flaviviruses. Compelling experimental evidence indicates each duplicated cassette to play a different role in viral replication. An association between the duplication of structural elements and the capacity of the genome to replicate in mammalian and arthropod hosts has been established (for review see Villordo et al., 2016).

**FIGURE 4:**
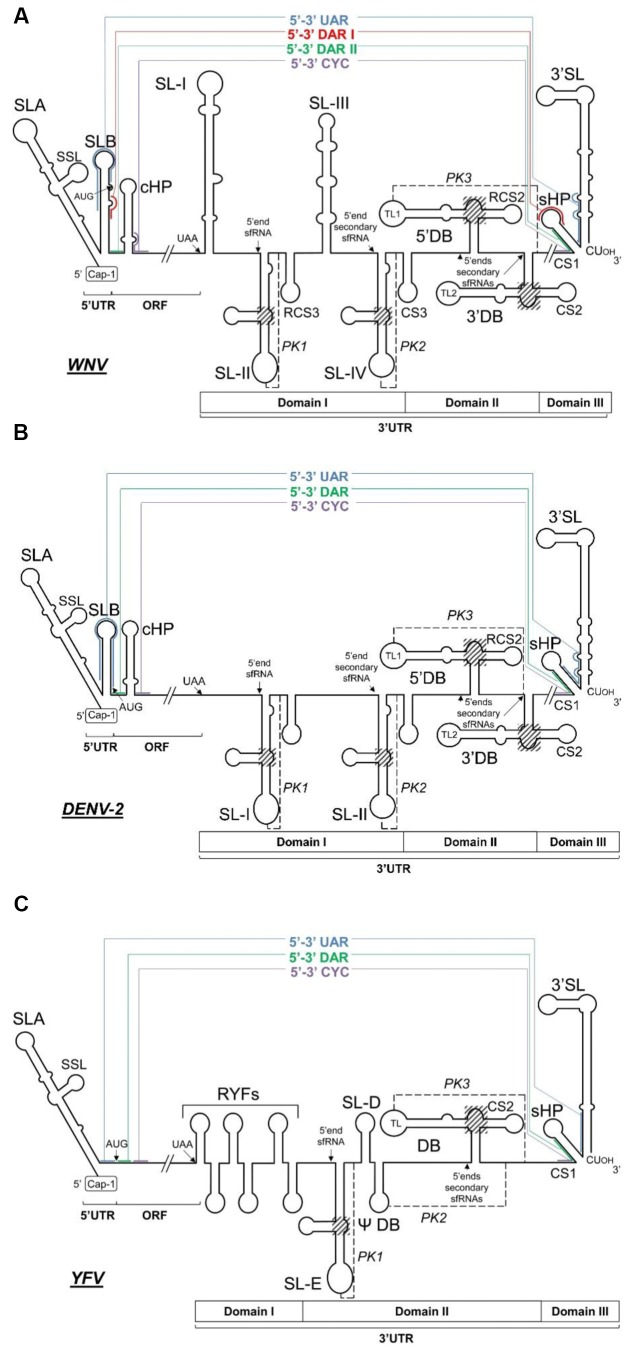
**Long-range RNA–RNA contacts in representative flavivirus RNA linear genomes**. Diagrams show the proposed conserved secondary structural elements and sequence motifs within the 5′ and the 3′ ends of three representative MBFV genomes, **(A)** WNV, **(B)** DENV-2, and **(C)** YFV. The ORF and the 5′ and 3′UTRs are indicated. Thin colored lines denote long-distance RNA–RNA interactions between genomic termini; the interacting sequences are shown by solid colored lines. Translation start and stop codons and the 5′ end sites of the subgenomic flavivirus RNAs (sfRNAs) are indicated by arrows. The three-way junctions critical for the generation of the sfRNAs are included in shadowed boxes. The pseudoknot elements (PK1, PK2, and PK3) are indicated by black dashed lines.

Domain I is located just downstream of the translation stop codon. In most flaviviruses it appears as a hypervariable sequence followed by two conserved stem-loop domains (SL-I and -II) similar in sequence and structure (**Figures 3**, **4**); in YFV (Wang et al., 1996), the NKV flaviviruses (Leyssen et al., 2002), and ISFVs [for a review see (Blitvich and Firth, 2015)], however, there is only one stem-loop (SL). In YFV, domain I contains tandem repeats in hairpin structures (RYFs) unique to the ISFV group (Bryant et al., 2005). The SL of the NKV flaviviruses is similar to those of the TBFVs (Villordo et al., 2016), while differences are observed in the structure of this region within the two main subgroups of the ISFVs – classical ISFVs (cISFV) and dual-host affiliated ISFVs (dISFV) (Blitvich and Firth, 2015). Although all ISFVs contain multiple sequence repeats (Gritsun et al., 2014), cISFVs are characterized by folding into short hairpins, and the dISFVs into an SL similar to those seen in MBFVs (Villordo et al., 2016). Domain I of the prototypical DENV-2 comprises a duplicated SL preceded by the hypervariable tract. The nucleotides of the apical loop of both SLs are involved in the formation of pseudoknots with the nearby downstream sequence (forming PK1 and PK2) (Thurner et al., 2004). In the JEV group (**Table 1**), the hypervariable region folds into an AU-rich stem-loop (SL-I) followed by a highly conserved branched element (SL-II) immediately preceded by a short conserved hairpin (RCS3) (Brinton, 2014) (**Figure 3**). This structural unit (SL-I•SL-II•RCS3) is repeated to yield the SL-III, SL-IV, and CS3 elements (Proutski et al., 1999) (**Figure 3**). Deletion and sequence mutation analyses of SL-I and II, and of the motifs RCS3 and CS3, have revealed their roles as regulatory replication elements (Lo et al., 2003; Pijlman et al., 2008). Importantly, the apical loop of SL-II is involved in the formation of a pseudoknot structure (PK1) with the single stranded region immediately downstream (**Figures 3**, **4**). The formation of this PK is critical for infectivity (Lo et al., 2003; Pijlman et al., 2008). A second pseudoknot, PK2, is formed in the repeated structural unit SL-III•SL-IV•CS3. Interestingly, several GNRA-like motifs are found in domain I, suggesting this region to function as a protein recruiting platform and as a nucleation center for direct RNA–RNA interactions (Sztuba-Solinska et al., 2013). TBFV duplicated stem-loops are Y-shaped (Gritsun and Gould, 2007a) – a different type of folding than seen in MBFV genomes. It is remarkable that their involvement in PK formation with downstream sequences is preserved, emphasizing the functional significance of the PK structural element.

Domain II is moderately conserved and in MBFV and NKV flaviviruses contains a characteristic structure known as a dumbbell (DB); this is involved in the formation of a PK structural element (**Figures 3**, **4**). In the DENV and JEV groups (**Table 1**), it contains a sequence motif duplicated in tandem (RCS2 and CS2) that forms an essential component of the respective functional dumbbell structures 5′DB and 3′DB (**Figure 3**) (Shurtleff et al., 2001; Silva et al., 2008). YFVs contain a pseudo-dumbbell (ψ-DB) which may be derived from a duplicated DB. In DENV, a pseudoknot structure (PK) has been proposed which involves the highly conserved 5 nt-long motif in the apical loop (top loop, TL1) of the 5′ hairpin in the 5′DB element plus the corresponding downstream single-stranded complementary sequence (Olsthoorn and Bol, 2001; Sztuba-Solinska et al., 2013). This structural element is likely to be formed in WNV as well (PK3, **Figure 3**). This architecture is functionally important for viral replication (Men et al., 1996; Lo et al., 2003; Alvarez et al., 2005), translation (Wei et al., 2009; Manzano et al., 2011) and infectivity (Proutski et al., 1999). NKV flaviviruses and dISFVs contain a single copy of the DB structure but there is no evidence of its involvement in a PK structure. In contrast TBFVs have no DB structure in this region, although they do have two different SLs. The more proximal, which is often duplicated, is known as GC-SL since its loop has a conserved GGC stretch involved in the formation of a PK element. The distal SL, AU-SL, is very stable and its loop contains a conserved AAUU sequence that participates in the formation of a second PK (Villordo et al., 2016).

Domain III is defined by the highly conserved terminal genomic functional elements sHP (short hairpin) and 3′SL (**Figures 3**, **4**). The presence of both has been confirmed by chemical probing (Brinton et al., 1986; Shi et al., 1996), SHAPE (selective 2′-hydroxyl acylation analyzed by primer extension) (Sztuba-Solinska et al., 2013) and nuclear magnetic resonance (NMR) analysis (Davis et al., 2013). The sHP element of domain III consists of a 5 bp stem and a highly conserved 6 nt apical loop (Brinton et al., 1986; Olsthoorn and Bol, 2001) that resembles the typical GN_N_RA motif. This suggests sHP to be a potential recruitment region of protein factors or to be involved in the establishment of RNA–RNA interactions. Partially overlapping with this sHP element, a highly conserved 24 nt-long sequence (CS1, **Figure 3**) has been shown indispensable for virus replication in DENV (Men et al., 1996). CS1 contains sequences involved in genome cyclization (Wengler and Castle, 1986; Hahn et al., 1987) (see below). The functional requirement of the CS1 nucleotides not involved in cyclization has not been explained. The terminal 3′SL is an essential structural element with a small number of conserved sequence stretches: the terminal 5′-CU_OH_-3′ and surrounding residues (Wengler, 1981; Brinton et al., 1986; Wengler and Castle, 1986) and the apical loop (Elghonemy et al., 2005; Tilgner et al., 2005) (**Figure 3**). In addition, all flaviviruses show a bulge in the upper portion of the 3′SL stem (Yu and Markoff, 2005) (**Figures 3**, **4**). This bulge induces a bend in the duplex, which might be required for NS5 protein recognition. The functions of sHP and 3′SL have been studied in depth, and are essential for viral replication (Blackwell and Brinton, 1995; Men et al., 1996; Khromykh and Westaway, 1997; Zeng et al., 1998; Bredenbeek et al., 2003; Villordo et al., 2010; Villordo and Gamarnik, 2013) and the completion of the viral cycle (Brinton et al., 1986; Hahn et al., 1987; Zeng et al., 1998; Khromykh et al., 2001; Alvarez et al., 2005; Tilgner et al., 2005; Yu and Markoff, 2005; Yu et al., 2008). They perform their functions likely by interacting with non-structural viral proteins (Chen et al., 1997a) and cellular factors such as eEF1A (Blackwell and Brinton, 1997; Davis et al., 2013), the La autoantigen (De Nova-Ocampo et al., 2002) and PTB (polypyrimidine tract binding protein) (De Nova-Ocampo et al., 2002). The role of 3′SL during viral translation initiation has also been widely studied, but the results obtained have been discrepant (Li and Brinton, 2001; Holden and Harris, 2004; Alvarez et al., 2005). Since flaviviruses do not bear a poly(A) tail, it has been proposed that 3′SL contributes to the recruitment of poly(A) tail binding protein (PABP) (Polacek et al., 2009b), and subsequently to ribosome recruitment and assembly. Finally, orthologous domains in DENV might be related to disease outcome (Mangada and Igarashi, 1997; Leitmeyer et al., 1999), suggesting a role for 3′ structural domains in virulence.

## Subgenomic Flavivirus RNAs

In addition to the accumulation of genomic RNA during flavivirus infection, subgenomic, non-coding flavivirus RNA molecules (sfRNAs) ranging from 300 to 500 nt-long accumulate in the cytoplasm (Urosevic et al., 1997; Lin et al., 2004; Pijlman et al., 2008). These molecules are the result of incomplete digestion of the viral genome by the host cell 5′–3′ exoribonuclease Xrn1, which cleaves the viral RNA but stalls at defined locations in the highly folded 3′UTR (Pijlman et al., 2008) (**Figure 4**). This resistance to Xrn1 activity is dependent on specific residues; these have been elucidated for WNV (Pijlman et al., 2008; Funk et al., 2010), YFV (Silva et al., 2010), DENV-2 (Chapman et al., 2014b), and MVEV (Chapman et al., 2014a), and are confirmed to be conserved across flaviviruses (Chapman et al., 2014a). Such residues share a common structural environment defined by a three-way junction and a characteristic and conserved pseudoknot, PK1 (Pijlman et al., 2008; Chapman et al., 2014b) (**Figures 3**, **4**), which is essential for sfRNA generation. In the DENV and JEV groups (**Table 1**), this structure has been located within the SL-I and SL-II of Domain I of the 3′UTR, respectively (Pijlman et al., 2008; Funk et al., 2010) (**Figures 4A,B**), while in YFV the single SL (SL-E) provides the stalling point (Silva et al., 2010) (**Figure 4C**). In MBFVs, it has been reported that the abrogation of PK1 leads to the production of shorter species of sfRNAs derived from the Xrn1 stalling at the downstream pseudoknot structures PK2 [SL-II in DENV-2 (**Figure 4B**), SL-IV in JEV (**Figure 4A**) and ψ-DB in YFV (**Figure 4C**)] and/or PK3 [5′DB in JEV and DENV and DB in YFV (**Figure 4**)] (Pijlman et al., 2008; Funk et al., 2010; Chapman et al., 2014a). Consecutive pseudoknots therefore appear to act as security or check points to assess the production of sfRNAs. The three-way junction organizes the three-dimensional folding by bringing the basal stem and the 3′ apical loop of the structure close together, yielding a ring-like topology with the free 5′ end inside it, as determined by X-ray crystallography (Chapman et al., 2014a). Thus, rather than providing a simple unfolding mechanism, Xrn1 turns the ring inside-out to provide access to the susceptible residues at the 5′ end. This architecture may also be responsible for the selection of directionality during extension by viral polymerase (Chapman et al., 2014a).

From a functional point of view, full-length sfRNAs play important roles in regulating the switch between translation and replication during the infectious cycle (Lin et al., 2004). They promote cytopathic effects and pathogenicity in mice (Pijlman et al., 2008; Funk et al., 2010; Chapman et al., 2014a; Liu et al., 2014) and they disrupt the generation of a proper immune response at different levels, while shortened sfRNA species lead to attenuated viral forms. In particular, full-length sfRNAs inhibit the antiviral activity of IFN-α/β by an unknown mechanism (Schuessler et al., 2012), as well as that of the antiviral RNAi pathway, probably by acting as Dicer decoy substrates (Schnettler et al., 2012). Intrinsic to sfRNA formation, Xrn1 function is inhibited and, thus, endogenous mRNAs are accumulated (Moon et al., 2012). Moreover, DENV-2 sfRNA has been shown to interact with stress granules (Bidet and Garcia-Blanco, 2014). Detailed information on the roles of sfRNAs is provided in recent reviews (Roby et al., 2014; Clarke et al., 2015; Charley and Wilusz, 2016).

## Genomic Cyclization in Flavivirus

The acquisition of a circular conformation in viral RNA genomes is a successful strategy that provides important advantages in the completion of the infective cycle. First of all, it efficiently ensures the propagation of undamaged, full-length genomes (Hahn et al., 1987). Further, the initiation of protein synthesis and the replication process is governed by the establishment of a closed loop topology. Transitions between different steps of the viral cycles are directly dependent on the existence of complex networks of RNA–RNA contacts (Romero-López and Berzal-Herranz, 2013).

The acquisition of the circular topology is mediated by direct, long distance RNA–RNA interactions between different complementary sequence motifs at the 5′ and 3′ ends of the genome (**Figure 4**). Such interactions have been probed by psoralen/UV crosslinking assays (You et al., 2001), electrophoretic mobility shift assays (Alvarez et al., 2005; Zhang et al., 2008a), atomic force microscopy (Alvarez et al., 2005), and structure probing (Dong et al., 2008; Polacek et al., 2009a). Though some of the complementary sequence motifs involved in genome cyclization show low conservation rates across the flaviviruses, the circularization mechanism is ubiquitous and required for flaviviral propagation (Khromykh et al., 2001; Song et al., 2008).

**FIGURE 5:**
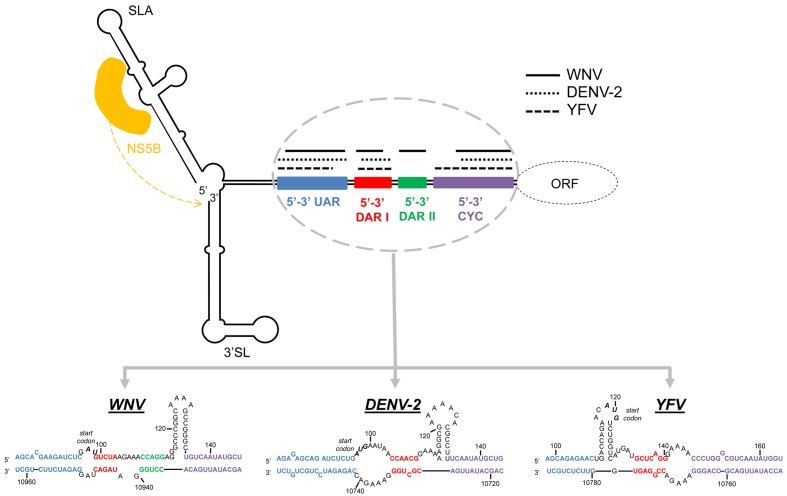
**Long-range RNA–RNA contacts in the circular flavivirus genome**. The diagram shows the circular conformation of representative flavivirus genomes mediated by long range RNA–RNA interactions. Colored boxes indicate the interacting sequences involved in genome cyclization. Lines above them represent their length in WNV (solid black lines), DENV-2 (dotted lines), and YFV (dashed lines). The sequence motifs within the 5′ and the 3′ ends are represented below the diagram for these MBFV models. Sequences correspond to the Kunjin virus MRM 61C strain (GenBank accession number L24511.1 for 5′UTR and L24512.1 for 3′UTR), the dengue virus serotype 2 (DENV-2) 16681 strain (GenBank accession number NC_001474) and the yellow fever virus (YFV) 17D vaccine strain (GenBank accession number X03700.1) respectively. The diagram also shows where the viral RdRp polymerase (yellow) binds in the genome ends.

In MBFV, at least three pairs of sequence motifs have been shown to participate in the cyclization process (**Figures 4**, **5**). These include:

(i) A highly conserved motif – the so-called 3′ cyclization sequence (3′CYC) – is included in the conserved sequence CS1 (**Figure 3**) just upstream of the sHP domain at the 3′ terminus of the genome. It contains an 8 nt-long stretch conserved across the MBFVs. The 3′CYC perfectly matches its complementary partner in the cHP domain at the extreme 5′ end (5′CYC) (Hahn et al., 1987) (**Figure 2**). The 5′–3′CYC interaction (**Figure 4**) must be preserved for efficient virus replication (Khromykh et al., 2001; Corver et al., 2003; Lo et al., 2003; Kofler et al., 2006). Different studies have reported sequence preferences in the 5′–3′CYC pairs (Suzuki et al., 2008; Basu and Brinton, 2011). Flipping specific base pairs can have different effects on virus replication depending on their position within the interacting domain. Mutations affecting the central positions of the CYC sequence, but with maintained base pairing at the points of 3′–5′ interaction, have little or no effect on replication, whereas base pairs flipped in the terminal positions severely affect viral replication. The role of the terminal and flanking CYC residues seems to be critical for initiating the interaction between the complementary sequences and for the preservation of the stability of the replication competent circular form.
(ii) The UAR pair, which involves residues upstream of the AUG start codon at the 5′ end of the viral genome, 5′UAR, and a complementary sequence located within the basal portion of the stem in the 3′SL element, 3′UAR (Alvarez et al., 2005; Zhang et al., 2008c) (**Figures 2–4**). It has been suggested that switching from the formation of the stem to the long-distance interaction with the 5′UAR releases the 3′ terminus of the viral genome for recognition by the flaviviral RNA polymerase (NS5) during the initiation of the minus-strand RNA synthesis (Zhang et al., 2008c; Polacek et al., 2009a; Filomatori et al., 2011; Davis et al., 2013) (**Figure 5**).
(iii) The DAR sequences motifs. In the DENV group, a single sequence motif 5′DAR within the linker between the SLB and cHP stems (at the 5′ end of the genome) interacts with the corresponding complementary 3′DAR sequence (included in the CS1 sequence) within the sHP stem at the genome 3′ terminus. In the JEV group (**Table 1**), two DAR motifs have been described – 5′DAR I and 5′DAR II – within the stem and the base of the SLB domain, which interact, respectively, with 3′DARI and 3′DARII (Dong et al., 2008; Friebe and Harris, 2010; Friebe et al., 2011) (**Figures 2–5**). During the initiation of minus-strand RNA synthesis, NS5 first recognizes the SLA element and the 5′DARII in the context of a circular RNA, and interacts with 3′DARI and II, probably leading to the initiation of viral replication (Dong et al., 2008) (**Figure 5**). These findings suggest a role for protein recruitment in DAR interactions and the subsequent genome cyclization process.

Data derived from structural, phylogenetic and functional analyses have allowed a theoretical model of the genomic cyclization process to be proposed (Friebe et al., 2011). Accordingly, the latter is initiated via the interaction between the 5′ and the 3′CYC motifs (Polacek et al., 2009a). The duplex then further extends via the DAR contacts which “open” the sHP element (Friebe and Harris, 2010; Friebe et al., 2011). Additional UAR-mediated interactions help to unwind the basal portion of the 3′SL domain to further promote conformational rearrangements within the 3′ end of the viral genome. Recently, a *cis*-acting element present in the capsid coding sequence of DENV was found to interact with 5′DB at the 3′UTR, forming a PK structural element. This interaction was proven to have a different effect on viral RNA replication in mosquito and mammalian cells (de Borba et al., 2015).

Tick-borne genomic cyclization occurs by the formation of at least the two long-distance interactions 5′–3′CSA and 5′–3′CSB (Mandl et al., 1993; Khromykh et al., 2001). The sequence motifs involved in these interactions are unrelated to those in MDFVs. The 5′–3′CSA interaction is the equivalent of the 5′–3′UAR interaction in MBFV, despite being located at different positions (Mandl et al., 1993) and is also crucial for RNA synthesis (Khromykh et al., 2001). The 5′CSB and 3′CSB motifs are located at genomic positions similar to 5′CYC and 3′CYC in MBFVs, but their interaction is not essential in TBFV replication (Kofler et al., 2006). Genome circularization in TBFVs is also enhanced by a kissing-loop contact involving two stem-loops, 5′SL6 and 3′SL3, located in the capsid coding region at the 5′ and 3′ ends, respectively (Tsetsarkin et al., 2016). The 5′SL6 domain was previously shown to be required for efficient replication (Tuplin et al., 2011).

In NKV flaviviruses, two interactions have been predicted involved in genome cyclization. The first involves a sequence motif located upstream of the AUG start codon and a complementary one within the 3′SL; the second is established between a motif within the capsid coding region and the corresponding counterpart upstream of the 3′SL (Leyssen et al., 2002).

In addition to the RNA–RNA interactions, genomic cyclization might be stabilized by viral and host protein factors recruited by different genomic RNA structural domains (Blackwell and Brinton, 1997; Ta and Vrati, 2000; De Nova-Ocampo et al., 2002; Garcia-Montalvo et al., 2004). These factors include the La protein (De Nova-Ocampo et al., 2002; Vashist et al., 2009), polypyrimidine-tract binding protein (PTB) (De Nova-Ocampo et al., 2002; Kim and Jeong, 2006) or translation elongation factor 1α (eEF-1α) (De Nova-Ocampo et al., 2002). Interestingly, such proteins are involved, at different extent, with the progression of the translation process, which points to cyclization as a feasible strategy to control viral protein synthesis. Different RNA helicases as FBP1 (far upstream element-binding protein), DDX3, DDX5, and DDX6 have also been proved to bind to both the 5′ and the 3′UTRs of the flavivirus genome, and affect replication in opposite ways (Chien et al., 2011; Ward et al., 2011; Li et al., 2013, 2014). These findings demonstrate that the control of the cyclization event is mediated by the RNA recruitment of host factors. They also show that flaviviruses can use the genome cyclation for regulating transitions between different steps of the infective cycle. Finally, host proteins related to mRNA splicing such as hnRNPA2 (Katoh et al., 2011) or Lsm1 (Dong et al., 2015) interact with the cyclization sequence motifs and/or with functional RNA domains located in the untranslated regions. The recruitment of these proteins is required for and efficient viral replication process though their molecular mechanism is still unknown.

A proper balance between linear and circular forms of the genome is required to ensure the initiation of plus-strand RNA synthesis, encapsidation, and even the switch from translation to replication. This is because sequences involved in the cyclization process overlap with essential structural domains that cannot be formed in the circular topology. In this context, the thermodynamic stability of the single structural domains is critical for efficient transition from one conformation to another. Thus, mutations that stabilize the circular or the linear form spontaneously revert to “less stable” architectures (Clyde et al., 2008; Villordo et al., 2010; Iglesias et al., 2011). The genome cyclization operates as a control system regulating the progression of the flavivirus infective cycle.

## Nucleic Acids Targeting Flavivirus Genomes

The information and functions encoded in structural genomic RNA domains render interference with the proper folding of these elements a candidate means of interfering with viral propagation. In this context, the use of nucleic acids as therapeutic agents is of growing interest. The development of any such therapy, however, must overcome a number of challenges, including the maintenance of the stability of the nucleic acid agents and efficient delivery to the target cell. These problems have been largely addressed by combinatorial chemistry, and a range of chemical nucleotide substitutions are now available. The use of chemically modified oligonucleotides has resulted in the improvement of the pharmacodynamic and pharmacokinetic properties of these antiviral nucleic acids-based antiviral agents (Haasnoot and Berkhout, 2009).

In recent decades, pioneering work into antisense oligonucleotide-based inhibitors has laid the ground for the design of thus-based antiviral compounds (Haasnoot and Berkhout, 2009). Antisense oligonucleotides are short nucleic acids with sequences complementary to those of their targets. They interfere with the function of essential regions within RNA molecules by different mechanisms. The first attempt to design antisense oligonucleotides against a flavivirus RNA genome used the DENV genome as a model (Raviprakash et al., 1995). A set of propynil-phosphorothioate-modified antisense oligonucleotides targeting five regions throughout the viral RNA showed that interfering with the sequence motif surrounding the translation initiation codon and the SL-IV domain within the 3′UTR was an effective antiviral strategy in cell culture.

These preliminary but promising results in DENV prompted further efforts to develop other antisense oligonucleotides against other flaviviruses, such as WNV. The use of phosphorodiamidate morpholino oligomers (PMOs) as potential anti-WNV drugs has been reported (Deas et al., 2005). Two oligonucleotides targeting the extreme 5′ end of the viral genome and the 3′CYC motif were found to efficiently interfere with viral translation and replication. Further, the conjugation of these PMOs at their 5′ end with an arginine-rich peptide (PPMO) improved uptake by cells, yielding an agent capable of strongly suppressing the viral cycle. It was suggested that the high conservation rate of the targeted regions allowed the design of sets of PPMOs targeting a spectrum of related flaviviruses belonging to the JEV group (Deas et al., 2007). This could lead to important advances in the use of nucleic acid-based compounds, not only as inhibitory molecules but also as biotechnological tools for the detection of different viruses in biological samples. In addition, modified PMOs could help us understand the molecular mechanisms underlying the function of the targeted structural RNA domains.

Cellular RNA interference (RNAi) has also been widely examined in recent years as a means of generating novel antiviral RNA molecules. This strategy is based on the design of short, double-stranded RNA molecules (the so-called small interfering RNAs or siRNAs), which are loaded into the RNA-induced silencing complex (RISC). The sense strand of the duplex then guides the complex to the target region, where it base-pairs fully to induce degradation of the target RNA molecule. As antisense oligonucleotides, siRNAs can be chemically produced or endogenously synthesized from appropriate expression vectors. Numerous authors have reported the use of siRNAs – both in cell culture and in infected mice – against the coding region of the WNV genome (Mccown et al., 2003; Bai et al., 2005; Geiss et al., 2005; Kumar et al., 2006; Ong et al., 2006, 2008; Yang et al., 2008) and the conserved functional domains within the 3′UTR (Zhang et al., 2008b; Anthony et al., 2009). The results confirm the potential of this strategy in the development of new antiviral compounds.

The use of RNA or DNA aptamers (short oligonucleotides that efficiently and specifically bind to a target molecule) represents another promising strategy for developing antiviral agents against flaviviruses. They also provide an interesting means of developing molecular tools for deciphering the functional role of genomic structural elements, and therefore the identification of potential therapeutic targets. This has already been shown for other, closely related viruses such as HCV (Marton et al., 2011, 2013; Fernández-Sanlés et al., 2015) as well as non-related viruses such as HIV (Sánchez-Luque et al., 2014). Aptamers can be chemically modified quite easily to increase their stability and improve their efficiency.

The successful clinical use of any of the above strategies is conditioned by the appearance of resistant mutants. In fact, WNV particles resistant to PMOs targeting the conserved 3′UAR sequence motif have already been isolated (Zhang et al., 2008b). They contained a single nucleotide mutation in the target sequence that impaired or weakened the PMO interaction, while the 5′UAR–3′UAR base-pairing was restored by the selection of a compensatory mutation. Novel strategies are therefore required, based on combining antiviral compounds with different specificities, including recognition of specific structural features, and even different mechanisms of action. In this context, the use of antisense oligonucleotides, siRNAs and other nucleic acid molecules (e.g., aptamers) in combination with other drugs, such as interferon or neutralizing antibodies, may provide effective and potent antiviral cocktails.

## Concluding Remarks

The acquisition of compact genomes was an important evolutionary achievement of RNA viruses; these genomes can store all the information required for the completion of the infectious cycle in reduced packages. This is possible due to the existence of a supracoding system beyond the nucleotide sequence, defined by discrete, folded domains, and higher-order structures. These elements operate both alone and in combination to create complex networks of contacts that regulate multiple steps of the viral cycle, and to recruit host and viral factors. Understanding how host–virus interactions shape viral evolution will help to elucidate the factors that govern the emergence of new viruses and the expansion of already known RNA viral pathogens. The lack of technics or experimental approaches to determine the RNA structure and to analyze the kinetics of RNA–RNA interactions in cell culture, together with the lack of experimental strategies to specifically interfere with the folding of the RNA genomic elements, represent an important limitation for understanding their function in the viral cycle. Importantly, the phylogenetic conservation of the genomic RNA structural domains and their interactions across members of *Flavivirus*, provide alternative and complementary potential targets to the viral proteins for novel antiviral compounds. Advances made in the field of nucleic acid synthesis have provided excellent candidate molecules for fighting RNA viruses by interfering with the essential functions performed by their genomic functional domains. Different pharmaceutical companies are now investigating the potential of nucleic acid therapeutic strategies, assessing long-term antiviral responses and trying to minimize secondary effects.

## Author Contributions

All authors participated in writing the manuscript (led by CR-L and AB-H), commented upon, and approved its final version. AF-S and PR-M prepared the figures.

## Conflict of Interest Statement

The authors declare that the research was conducted in the absence of any commercial or financial relationships that could be construed as a potential conflict of interest.

## Footnotes

1 http://www.who.int/mediacentre/en/

2 http://www.cdc.gov/

## References

[B1] Agis-Juarez R. A. Galvan I. Medina F. Daikoku T. Padmanabhan R. Ludert J. E. (2009). Polypyrimidine tract-binding protein is relocated to the cytoplasm and is required during dengue virus infection in Vero cells. *J. Gen. Virol.* 90 2893–2901. 10.1099/vir.0.013433-0 19692542

[B2] Allison S. L. Schalich J. Stiasny K. Mandl C. W. Kunz C. Heinz F. X. (1995). Oligomeric rearrangement of tick-borne encephalitis virus envelope proteins induced by an acidic pH. *J. Virol.* 69 695–700. 7529335 10.1128/jvi.69.2.695-700.1995 PMC188630

[B3] Alvarez D. E. De Lella Ezcurra A. L. Fucito S. Gamarnik A. V. (2005). Role of RNA structures present at the 3′UTR of dengue virus on translation, RNA synthesis, and viral replication. *Virology* 339 200–212. 10.1016/j.virol.2005.06.009 16002117

[B4] Anthony K. G. Bai F. Krishnan M. N. Fikrig E. Koski R. A. (2009). Effective siRNA targeting of the 3′ untranslated region of the West Nile virus genome. *Antiviral Res.* 82 166–168. 10.1016/j.antiviral.2008.12.007 19135091

[B5] Anwar A. Leong K. M. Ng M. L. Chu J. J. Garcia-Blanco M. A. (2009). The polypyrimidine tract-binding protein is required for efficient dengue virus propagation and associates with the viral replication machinery. *J. Biol. Chem.* 284 17021–17029. 10.1074/jbc.M109.006239 19380576 PMC2719340

[B6] Bai F. Wang T. Pal U. Bao F. Gould L. H. Fikrig E. (2005). Use of RNA interference to prevent lethal murine west nile virus infection. *J. Infect. Dis.* 191 1148–1154. 10.1086/428507 15747251

[B7] Basu M. Brinton M. A. (2011). West Nile virus (WNV) genome RNAs with up to three adjacent mutations that disrupt long distance 5′-3′ cyclization sequence basepairs are viable. *Virology* 412 220–232. 10.1016/j.virol.2011.01.008 21292293 PMC3056923

[B8] Bera A. K. Kuhn R. J. Smith J. L. (2007). Functional characterization of *cis* and *trans* activity of the flavivirus NS2B-NS3 protease. *J. Biol. Chem.* 282 12883–12892. 10.1074/jbc.M611318200 17337448

[B9] Bidet K. Garcia-Blanco M. A. (2014). Flaviviral RNAs: weapons and targets in the war between virus and host. *Biochem. J.* 462 215–230. 10.1042/BJ20140456 25102029

[B10] Blackwell J. L. Brinton M. A. (1995). BHK cell proteins that bind to the 3′ stem-loop structure of the West Nile virus genome RNA. *J. Virol.* 69 5650–5658. 7637011 10.1128/jvi.69.9.5650-5658.1995 PMC189422

[B11] Blackwell J. L. Brinton M. A. (1997). Translation elongation factor-1 alpha interacts with the 3′ stem-loop region of West Nile virus genomic RNA. *J. Virol.* 71 6433–6444. 9261361 10.1128/jvi.71.9.6433-6444.1997 PMC191917

[B12] Blitvich B. J. Firth A. E. (2015). Insect-specific flaviviruses: a systematic review of their discovery, host range, mode of transmission, superinfection exclusion potential and genomic organization. *Viruses* 7 1927–1959. 10.3390/v7041927 25866904 PMC4411683

[B13] Bogachek M. V. Zaitsev B. N. Sekatskii S. K. Protopopova E. V. Ternovoi V. A. Ivanova A. V. (2010). Characterization of glycoprotein E C-end of West Nile virus and evaluation of its interaction force with alphaVbeta3 integrin as putative cellular receptor. *Biochemistry* 75 472–480. 20618137 10.1134/s0006297910040115

[B14] Bredenbeek P. J. Kooi E. A. Lindenbach B. Huijkman N. Rice C. M. Spaan W. J. (2003). A stable full-length yellow fever virus cDNA clone and the role of conserved RNA elements in flavivirus replication. *J. Gen. Virol.* 84 1261–1268. 10.1099/vir.0.18860-0 12692292

[B15] Brinton M. A. (2001). Host factors involved in West Nile virus replication. *Ann. N. Y. Acad. Sci.* 951 207–219. 10.1111/j.1749-6632.2001.tb02698.x 11797778

[B16] Brinton M. A. (2002). The molecular biology of West Nile Virus: a new invader of the western hemisphere. *Annu. Rev. Microbiol.* 56 371–402. 10.1146/annurev.micro.56.012302.160654 12142476

[B17] Brinton M. A. (2014). Replication cycle and molecular biology of the West Nile virus. *Viruses* 6 13–53. 10.3390/v6010013 PMC3917430 24378320

[B18] Brinton M. A. Dispoto J. H. (1988). Sequence and secondary structure analysis of the 5′-terminal region of flavivirus genome RNA. *Virology* 162 290–299. 10.1016/0042-6822(88)90468-0 2829420

[B19] Brinton M. A. Fernandez A. V. Dispoto J. H. (1986). The 3′-nucleotides of flavivirus genomic RNA form a conserved secondary structure. *Virology* 153 113–121. 10.1016/0042-6822(86)90012-7 3016981

[B20] Bryant J. E. Vasconcelos P. F. Rijnbrand R. C. Mutebi J. P. Higgs S. Barrett A. D. (2005). Size heterogeneity in the 3′ noncoding region of South American isolates of yellow fever virus. *J. Virol.* 79 3807–3821. 10.1128/JVI.79.6.3807-3821.2005 15731274 PMC1075708

[B21] Cahour A. Pletnev A. Vazielle-Falcoz M. Rosen L. Lai C. J. (1995). Growth-restricted dengue virus mutants containing deletions in the 5′ noncoding region of the RNA genome. *Virology* 207 68–76. 10.1006/viro.1995.1052 7871753

[B22] Castle E. Nowak T. Leidner U. Wengler G. (1985). Sequence analysis of the viral core protein and the membrane-associated proteins V1 and NV2 of the flavivirus West Nile virus and of the genome sequence for these proteins. *Virology* 145 227–236. 10.1016/0042-6822(85)90156-4 2992152

[B23] Castle E. Wengler G. (1987). Nucleotide sequence of the 5′-terminal untranslated part of the genome of the flavivirus West Nile virus. *Arch. Virol.* 92 309–313. 10.1007/BF01317487 3813889

[B24] Chapman E. G. Costantino D. A. Rabe J. L. Moon S. L. Wilusz J. Nix J. C. (2014a). The structural basis of pathogenic subgenomic flavivirus RNA (sfRNA) production. *Science* 344 307–310. 10.1126/science.1250897 24744377 PMC4163914

[B25] Chapman E. G. Moon S. L. Wilusz J. Kieft J. S. (2014b). RNA structures that resist degradation by Xrn1 produce a pathogenic dengue virus RNA. *Elife* 3:e01892 10.7554/eLife.01892 PMC3968743 24692447

[B26] Charley P. A. Wilusz J. (2016). Standing your ground to exoribonucleases: function of Flavivirus long non-coding RNAs. *Virus Res.* 212 70–77. 10.1016/j.virusres.2015.09.009 26368052 PMC4744573

[B27] Chen C. J. Kuo M. D. Chien L. J. Hsu S. L. Wang Y. M. Lin J. H. (1997a). RNA-protein interactions: involvement of NS3, NS5, and 3′ noncoding regions of Japanese encephalitis virus genomic RNA. *J. Virol.* 71 3466–3473. 9094618 10.1128/jvi.71.5.3466-3473.1997 PMC191493

[B28] Chen Y. Maguire T. Hileman R. E. Fromm J. R. Esko J. D. Linhardt R. J. (1997b). Dengue virus infectivity depends on envelope protein binding to target cell heparan sulfate. *Nat. Med.* 3 866–871. 9256277 10.1038/nm0897-866

[B29] Chien H. L. Liao C. L. Lin Y. L. (2011). FUSE binding protein 1 interacts with untranslated regions of Japanese encephalitis virus RNA and negatively regulates viral replication. *J. Virol.* 85 4698–4706. 10.1128/JVI.01950-10 21367899 PMC3126168

[B30] Chu J. J. Ng M. L. (2004). Infectious entry of West Nile virus occurs through a clathrin-mediated endocytic pathway. *J. Virol.* 78 10543–10555. 10.1128/JVI.78.19.10543-10555.2004 15367621 PMC516396

[B31] Clarke B. D. Roby J. A. Slonchak A. Khromykh A. A. (2015). Functional non-coding RNAs derived from the flavivirus 3′ untranslated region. *Virus Res.* 206 53–61. 10.1016/j.virusres.2015.01.026 25660582

[B32] Clyde K. Barrera J. Harris E. (2008). The capsid-coding region hairpin element (cHP) is a critical determinant of dengue virus and West Nile virus RNA synthesis. *Virology* 379 314–323. 10.1016/j.virol.2008.06.034 18676000 PMC2628549

[B33] Clyde K. Harris E. (2006). RNA secondary structure in the coding region of dengue virus type 2 directs translation start codon selection and is required for viral replication. *J. Virol.* 80 2170–2182. 10.1128/JVI.80.5.2170-2182.2006 16474125 PMC1395379

[B34] Cook S. Holmes E. C. (2006). A multigene analysis of the phylogenetic relationships among the flaviviruses (Family: Flaviviridae) and the evolution of vector transmission. *Arch. Virol.* 151 309–325. 10.1007/s00705-005-0626-6 16172840

[B35] Cook S. Moureau G. Kitchen A. Gould E. A. De Lamballerie X. Holmes E. C. (2012). Molecular evolution of the insect-specific flaviviruses. *J. Gen. Virol.* 93 223–234. 10.1099/vir.0.036525-0 22012464 PMC3352342

[B36] Corver J. Lenches E. Smith K. Robison R. A. Sando T. Strauss E. G. (2003). Fine mapping of a *cis*-acting sequence element in yellow fever virus RNA that is required for RNA replication and cyclization. *J. Virol.* 77 2265–2270. 10.1128/JVI.77.3.2265-2270.2003 12525663 PMC140906

[B37] Davis C. W. Nguyen H. Y. Hanna S. L. Sanchez M. D. Doms R. W. Pierson T. C. (2006). West Nile virus discriminates between DC-SIGN and DC-SIGNR for cellular attachment and infection. *J. Virol.* 80 1290–1301. 10.1128/JVI.80.3.1290-1301.2006 16415006 PMC1346927

[B38] Davis W. G. Basu M. Elrod E. J. Germann M. W. Brinton M. A. (2013). Identification of *cis*-acting nucleotides and a structural feature in West Nile virus 3′-terminus RNA that facilitate viral minus strand RNA synthesis. *J. Virol.* 87 7622–7636. 10.1128/JVI.00212-13 23637406 PMC3700269

[B39] de Borba L. Villordo S. M. Iglesias N. G. Filomatori C. V. Gebhard L. G. Gamarnik A. V. (2015). Overlapping local and long-range RNA-RNA interactions modulate dengue virus genome cyclization and replication. *J. Virol.* 89 3430–3437. 10.1128/JVI.02677-14 25589642 PMC4337542

[B40] de Lamballerie X. Crochu S. Billoir F. Neyts J. De Micco P. Holmes E. C. (2002). Genome sequence analysis of Tamana bat virus and its relationship with the genus Flavivirus. *J. Gen. Virol.* 83 2443–2454. 10.1099/0022-1317-83-10-2443 12237426

[B41] De Nova-Ocampo M. Villegas-Sepulveda N. Del Angel R. M. (2002). Translation elongation factor-1alpha, La, and PTB interact with the 3′ untranslated region of dengue 4 virus RNA. *Virology* 295 337–347. 10.1006/viro.2002.1407 12033793

[B42] Deas T. S. Bennett C. J. Jones S. A. Tilgner M. Ren P. Behr M. J. (2007). In vitro resistance selection and in vivo efficacy of morpholino oligomers against West Nile virus. *Antimicrob. Agents Chemother.* 51 2470–2482. 10.1128/AAC.00069-07 17485503 PMC1913242

[B43] Deas T. S. Binduga-Gajewska I. Tilgner M. Ren P. Stein D. A. Moulton H. M. (2005). Inhibition of flavivirus infections by antisense oligomers specifically suppressing viral translation and RNA replication. *J. Virol.* 79 4599–4609. 10.1128/JVI.79.8.4599-4609.2005 15795246 PMC1069577

[B44] Dong H. Zhang B. Shi P. Y. (2008). Terminal structures of West Nile virus genomic RNA and their interactions with viral NS5 protein. *Virology* 381 123–135. 10.1016/j.virol.2008.07.040 18799181

[B45] Dong Y. Yang J. Ye W. Wang Y. Miao Y. Ding T. (2015). LSm1 binds to the Dengue virus RNA 3′ UTR and is a positive regulator of Dengue virus replication. *Int. J. Mol. Med.* 35 1683–1689. 10.3892/ijmm.2015.2169 25872476

[B46] Edgil D. Polacek C. Harris E. (2006). Dengue virus utilizes a novel strategy for translation initiation when cap-dependent translation is inhibited. *J. Virol.* 80 2976–2986. 10.1128/JVI.80.6.2976-2986.2006 16501107 PMC1395423

[B47] Eigen M. (2002). Error catastrophe and antiviral strategy. *Proc. Natl. Acad. Sci. U.S.A.* 99 13374–13376. 10.1073/pnas.212514799 12370416 PMC129678

[B48] Elghonemy S. Davis W. G. Brinton M. A. (2005). The majority of the nucleotides in the top loop of the genomic 3′ terminal stem loop structure are *cis*-acting in a West Nile virus infectious clone. *Virology* 331 238–246. 10.1016/j.virol.2004.11.008 15629768

[B49] Emara M. M. Brinton M. A. (2007). Interaction of TIA-1/TIAR with West Nile and dengue virus products in infected cells interferes with stress granule formation and processing body assembly. *Proc. Natl. Acad. Sci. U.S.A.* 104 9041–9046. 10.1073/pnas.0703348104 17502609 PMC1885624

[B50] Fernández-Sanlés A. Berzal-Herranz B. González-Matamala R. Ríos-Marco P. Romero-López C. Berzal-Herranz A. (2015). RNA Aptamers as molecular tools to study the functionality of the hepatitis C virus CRE region. *Molecules* 20 16030–16047. 10.3390/molecules200916030 26364632 PMC6331917

[B51] Filomatori C. V. Iglesias N. G. Villordo S. M. Alvarez D. E. Gamarnik A. V. (2011). RNA sequences and structures required for the recruitment and activity of the dengue virus polymerase. *J. Biol. Chem.* 286 6929–6939. 10.1074/jbc.M110.162289 21183683 PMC3044948

[B52] Filomatori C. V. Lodeiro M. F. Alvarez D. E. Samsa M. M. Pietrasanta L. Gamarnik A. V. (2006). A 5′ RNA element promotes dengue virus RNA synthesis on a circular genome. *Genes Dev.* 20 2238–2249. 10.1101/gad.1444206 16882970 PMC1553207

[B53] Friebe P. Harris E. (2010). Interplay of RNA elements in the dengue virus 5′ and 3′ ends required for viral RNA replication. *J. Virol.* 84 6103–6118. 10.1128/JVI.02042-09 20357095 PMC2876622

[B54] Friebe P. Shi P. Y. Harris E. (2011). The 5′ and 3′ downstream AUG region elements are required for mosquito-borne flavivirus RNA replication. *J. Virol.* 85 1900–1905. 10.1128/JVI.02037-10 21123391 PMC3028882

[B55] Friedrich S. Schmidt T. Geissler R. Lilie H. Chabierski S. Ulbert S. (2014). AUF1 p45 promotes West Nile virus replication by an RNA chaperone activity that supports cyclization of the viral genome. *J. Virol.* 88 11586–11599. 10.1128/JVI.01283-14 25078689 PMC4178777

[B56] Funk A. Truong K. Nagasaki T. Torres S. Floden N. Balmori Melian E. (2010). RNA structures required for production of subgenomic flavivirus RNA. *J. Virol.* 84 11407–11417. 10.1128/JVI.01159-10 20719943 PMC2953152

[B57] Garcia-Montalvo B. M. Medina F. Del Angel R. M. (2004). La protein binds to NS5 and NS3 and to the 5′ and 3′ ends of dengue 4 virus RNA. *Virus Res.* 102 141–150. 10.1016/j.virusres.2004.01.024 15084396

[B58] Geiss B. J. Pierson T. C. Diamond M. S. (2005). Actively replicating West Nile virus is resistant to cytoplasmic delivery of siRNA. *Virol. J.* 2 53 10.1186/1743-422X-2-53 PMC1174879 15985182

[B59] Gillespie L. K. Hoenen A. Morgan G. Mackenzie J. M. (2010). The endoplasmic reticulum provides the membrane platform for biogenesis of the flavivirus replication complex. *J. Virol.* 84 10438–10447. 10.1128/JVI.00986-10 20686019 PMC2950591

[B60] Gritsun D. J. Jones I. M. Gould E. A. Gritsun T. S. (2014). Molecular archaeology of Flaviviridae untranslated regions: duplicated RNA structures in the replication enhancer of flaviviruses and pestiviruses emerged via convergent evolution. *PLoS ONE* 9:e92056 10.1371/journal.pone.0092056 PMC3960163 24647143

[B61] Gritsun T. S. Gould E. A. (2007a). Origin and evolution of 3′UTR of flaviviruses: long direct repeats as a basis for the formation of secondary structures and their significance for virus transmission. *Adv. Virus Res.* 69 203–248. 10.1016/S0065-3527(06)69005-2 17222695

[B62] Gritsun T. S. Gould E. A. (2007b). Origin and evolution of flavivirus 5′UTRs and panhandles: trans-terminal duplications? *Virology* 366 8–15. 10.1016/j.virol.2007.04.011 17658577

[B63] Gritsun T. S. Venugopal K. Zanotto P. M. Mikhailov M. V. Sall A. A. Holmes E. C. (1997). Complete sequence of two tick-borne flaviviruses isolated from Siberia and the UK: analysis and significance of the 5′ and 3′-UTRs. *Virus Res.* 49 27–39. 10.1016/S0168-1702(97)01451-2 9178494

[B64] Groat-Carmona A. M. Orozco S. Friebe P. Payne A. Kramer L. Harris E. (2012). A novel coding-region RNA element modulates infectious dengue virus particle production in both mammalian and mosquito cells and regulates viral replication in *Aedes aegypti* mosquitoes. *Virology* 432 511–526. 10.1016/j.virol.2012.06.028 22840606 PMC3683586

[B65] Haasnoot J. Berkhout B. (2009). Nucleic acids-based therapeutics in the battle against pathogenic viruses. *Handb. Exp. Pharmacol.* 189 243–263. 10.1007/978-3-540-79086-0_9 PMC7119910 19048203

[B66] Hahn C. S. Hahn Y. S. Rice C. M. Lee E. Dalgarno L. Strauss E. G. (1987). Conserved elements in the 3′ untranslated region of flavivirus RNAs and potential cyclization sequences. *J. Mol. Biol.* 198 33–41. 10.1016/0022-2836(87)90455-4 2828633

[B67] Hoenen A. Liu W. Kochs G. Khromykh A. A. Mackenzie J. M. (2007). West Nile virus-induced cytoplasmic membrane structures provide partial protection against the interferon-induced antiviral MxA protein. *J. Gen. Virol.* 88 3013–3017. 10.1099/vir.0.83125-0 17947524

[B68] Holden K. L. Harris E. (2004). Enhancement of dengue virus translation: role of the 3′ untranslated region and the terminal 3′ stem-loop domain. *Virology* 329 119–133. 10.1016/j.virol.2004.08.004 15476880

[B69] Iglesias N. G. Filomatori C. V. Gamarnik A. V. (2011). The F1 motif of dengue virus polymerase NS5 is involved in promoter-dependent RNA synthesis. *J. Virol.* 85 5745–5756. 10.1128/JVI.02343-10 21471248 PMC3126321

[B70] Katoh H. Mori Y. Kambara H. Abe T. Fukuhara T. Morita E. (2011). Heterogeneous nuclear ribonucleoprotein A2 participates in the replication of Japanese encephalitis virus through an interaction with viral proteins and RNA. *J. Virol.* 85 10976–10988. 10.1128/JVI.00846-11 21865391 PMC3194950

[B71] Kaufusi P. H. Kelley J. F. Yanagihara R. Nerurkar V. R. (2014). Induction of endoplasmic reticulum-derived replication-competent membrane structures by West Nile virus non-structural protein 4B. *PLoS ONE* 9:e84040 10.1371/journal.pone.0084040 PMC3896337 24465392

[B72] Khromykh A. A. Meka H. Guyatt K. J. Westaway E. G. (2001). Essential role of cyclization sequences in flavivirus RNA replication. *J. Virol.* 75 6719–6728. 10.1128/JVI.75.14.6719-6728.2001 11413342 PMC114398

[B73] Khromykh A. A. Westaway E. G. (1997). Subgenomic replicons of the flavivirus Kunjin: construction and applications. *J. Virol.* 71 1497–1505. 8995675 10.1128/jvi.71.2.1497-1505.1997 PMC191206

[B74] Kim S. M. Jeong Y. S. (2006). Polypyrimidine tract-binding protein interacts with the 3′ stem-loop region of Japanese encephalitis virus negative-strand RNA. *Virus Res.* 115 131–140. 10.1016/j.virusres.2005.07.013 16181699

[B75] Kofler R. M. Hoenninger V. M. Thurner C. Mandl C. W. (2006). Functional analysis of the tick-borne encephalitis virus cyclization elements indicates major differences between mosquito-borne and tick-borne flaviviruses. *J. Virol.* 80 4099–4113. 10.1128/JVI.80.8.4099-4113.2006 16571826 PMC1440478

[B76] Kozak M. (1990). Downstream secondary structure facilitates recognition of initiator codons by eukaryotic ribosomes. *Proc. Natl. Acad. Sci. U.S.A.* 87 8301–8305. 10.1073/pnas.87.21.8301 2236042 PMC54943

[B77] Kroschewski H. Allison S. L. Heinz F. X. Mandl C. W. (2003). Role of heparan sulfate for attachment and entry of tick-borne encephalitis virus. *Virology* 308 92–100. 10.1016/S0042-6822(02)00097-1 12706093

[B78] Kumar P. Lee S. K. Shankar P. Manjunath N. (2006). A single siRNA suppresses fatal encephalitis induced by two different flaviviruses. *PLoS Med.* 3:e96 10.1371/journal.pmed.0030096 PMC1361782 16464133

[B79] Kuno G. Chang G. J. Tsuchiya K. R. Karabatsos N. Cropp C. B. (1998). Phylogeny of the genus Flavivirus. *J. Virol.* 72 73–83. 9420202 10.1128/jvi.72.1.73-83.1998 PMC109351

[B80] Leitmeyer K. C. Vaughn D. W. Watts D. M. Salas R. Villalobos I. De C. (1999). Dengue virus structural differences that correlate with pathogenesis. *J. Virol.* 73 4738–4747. 10233934 10.1128/jvi.73.6.4738-4747.1999 PMC112516

[B81] Leyssen P. Charlier N. Lemey P. Billoir F. Vandamme A. M. De Clercq E. (2002). Complete genome sequence, taxonomic assignment, and comparative analysis of the untranslated regions of the Modoc virus, a flavivirus with no known vector. *Virology* 293 125–140. 10.1006/viro.2001.1241 11853406

[B82] Li C. Ge L. L. Li P. P. Wang Y. Dai J. J. Sun M. X. (2014). Cellular DDX3 regulates Japanese encephalitis virus replication by interacting with viral un-translated regions. *Virology* 449 70–81. 10.1016/j.virol.2013.11.008 24418539 PMC7111930

[B83] Li C. Ge L. L. Li P. P. Wang Y. Sun M. X. Huang L. (2013). The DEAD-box RNA helicase DDX5 acts as a positive regulator of Japanese encephalitis virus replication by binding to viral 3′. *UTR. Antiviral Res.* 100 487–499. 10.1016/j.antiviral.2013.09.002 24035833 PMC7113685

[B84] Li W. Brinton M. A. (2001). The 3′ stem loop of the West Nile virus genomic RNA can suppress translation of chimeric mRNAs. *Virology* 287 49–61. 10.1006/viro.2001.1015 11504541

[B85] Li W. Li Y. Kedersha N. Anderson P. Emara M. Swiderek K. M. (2002). Cell proteins TIA-1 and TIAR interact with the 3′ stem-loop of the West Nile virus complementary minus-strand RNA and facilitate virus replication. *J. Virol.* 76 11989–12000. 10.1128/JVI.76.23.11989-12000.2002 12414941 PMC136884

[B86] Li X. F. Jiang T. Yu X. D. Deng Y. Q. Zhao H. Zhu Q. Y. (2010). RNA elements within the 5′ untranslated region of the West Nile virus genome are critical for RNA synthesis and virus replication. *J. Gen. Virol.* 91 1218–1223. 10.1099/vir.0.013854-0 20016034

[B87] Lin K. C. Chang H. L. Chang R. Y. (2004). Accumulation of a 3′-terminal genome fragment in Japanese encephalitis virus-infected mammalian and mosquito cells. *J. Virol.* 78 5133–5138. 10.1128/JVI.78.10.5133-5138.2004 15113895 PMC400339

[B88] Liu Y. Liu H. Zou J. Zhang B. Yuan Z. (2014). Dengue virus subgenomic RNA induces apoptosis through the Bcl-2-mediated PI3k/Akt signaling pathway. *Virology* 448 15–25. 10.1016/j.virol.2013.09.016 24314632

[B89] Liu Z. Y. Li X. F. Jiang T. Deng Y. Q. Zhao H. Wang H. J. (2013). Novel *cis*-acting element within the capsid-coding region enhances flavivirus viral-RNA replication by regulating genome cyclization. *J. Virol.* 87 6804–6818. 10.1128/JVI.00243-13 23576500 PMC3676100

[B90] Lo M. K. Tilgner M. Bernard K. A. Shi P. Y. (2003). Functional analysis of mosquito-borne flavivirus conserved sequence elements within 3′ untranslated region of West Nile virus by use of a reporting replicon that differentiates between viral translation and RNA replication. *J. Virol.* 77 10004–10014. 10.1128/JVI.77.18.10004-10014.2003 12941911 PMC224605

[B91] Lodeiro M. F. Filomatori C. V. Gamarnik A. V. (2009). Structural and functional studies of the promoter element for dengue virus RNA replication. *J. Virol.* 83 993–1008. 10.1128/JVI.01647-08 19004935 PMC2612346

[B92] Mandl C. W. Holzmann H. Kunz C. Heinz F. X. (1993). Complete genomic sequence of Powassan virus: evaluation of genetic elements in tick-borne versus mosquito-borne flaviviruses. *Virology* 194 173–184. 10.1006/viro.1993.1247 8097605

[B93] Mandl C. W. Kunz C. Heinz F. X. (1991). Presence of poly(A) in a flavivirus: significant differences between the 3′ noncoding regions of the genomic RNAs of tick-borne encephalitis virus strains. *J. Virol.* 65 4070–4077. 1712858 10.1128/jvi.65.8.4070-4077.1991 PMC248839

[B94] Mangada M. N. Igarashi A. (1997). Sequences of terminal non-coding regions from four dengue-2 viruses isolated from patients exhibiting different disease severities. *Virus Genes* 14 5–12. 10.1023/A:1007914520454 9208450

[B95] Manzano M. Reichert E. D. Polo S. Falgout B. Kasprzak W. Shapiro B. A. (2011). Identification of *cis*-acting elements in the 3′-untranslated region of the dengue virus type 2 RNA that modulate translation and replication. *J. Biol. Chem.* 286 22521–22534. 10.1074/jbc.M111.234302 21515677 PMC3121397

[B96] Marton S. Berzal-Herranz B. Garmendia E. Cueto F. J. Berzal-Herranz A. (2011). Anti-HCV RNA aptamers targeting the genomic CRE element. *Pharmaceuticals* 5 49–60. 10.3390/ph5010049 24288042 PMC3763624

[B97] Marton S. Romero-López C. Berzal-Herranz A. (2013). RNA aptamer-mediated interference of HCV replication by targeting the CRE-5BSL3.2 *domain*. *J. Viral Hepat.* 20 103–112. 10.1111/j.1365-2893.2012.01629.x 23301545

[B98] Mccown M. Diamond M. S. Pekosz A. (2003). The utility of siRNA transcripts produced by RNA polymerase I in down regulating viral gene expression and replication of negative- and positive-strand RNA viruses. *Virology* 313 514–524. 10.1016/S0042-6822(03)00341-6 12954218

[B99] Medigeshi G. R. Hirsch A. J. Streblow D. N. Nikolich-Zugich J. Nelson J. A. (2008). West Nile virus entry requires cholesterol-rich membrane microdomains and is independent of alphavbeta3 integrin. *J. Virol.* 82 5212–5219. 10.1128/JVI.00008-08 18385233 PMC2395215

[B100] Men R. Bray M. Clark D. Chanock R. M. Lai C. J. (1996). Dengue type 4 virus mutants containing deletions in the 3′ noncoding region of the RNA genome: analysis of growth restriction in cell culture and altered viremia pattern and immunogenicity in rhesus monkeys. *J. Virol.* 70 3930–3937. 8648730 10.1128/jvi.70.6.3930-3937.1996 PMC190271

[B101] Merrick W. C. (2004). Cap-dependent and cap-independent translation in eukaryotic systems. *Gene* 332 1–11. 10.1016/j.gene.2004.02.051 15145049

[B102] Moon S. L. Anderson J. R. Kumagai Y. Wilusz C. J. Akira S. Khromykh A. A. (2012). A noncoding RNA produced by arthropod-borne flaviviruses inhibits the cellular exoribonuclease XRN1 and alters host mRNA stability. *RNA* 18 2029–2040. 10.1261/rna.034330.112 23006624 PMC3479393

[B103] Mukhopadhyay S. Kuhn R. J. Rossmann M. G. (2005). A structural perspective of the flavivirus life cycle. *Nat. Rev. Microbiol.* 3 13–22. 10.1038/nrmicro1067 15608696

[B104] Nowak T. Farber P. M. Wengler G. (1989). Analyses of the terminal sequences of West Nile virus structural proteins and of the in vitro translation of these proteins allow the proposal of a complete scheme of the proteolytic cleavages involved in their synthesis. *Virology* 169 365–376. 10.1016/0042-6822(89)90162-1 2705302

[B105] Olsthoorn R. C. Bol J. F. (2001). Sequence comparison and secondary structure analysis of the 3′ noncoding region of flavivirus genomes reveals multiple pseudoknots. *RNA* 7 1370–1377. 11680841 PMC1370180

[B106] Ong S. P. Choo B. G. Chu J. J. Ng M. L. (2006). Expression of vector-based small interfering RNA against West Nile virus effectively inhibits virus replication. *Antiviral Res.* 72 216–223. 10.1016/j.antiviral.2006.06.005 16870272

[B107] Ong S. P. Chu J. J. Ng M. L. (2008). Inhibition of West Nile virus replication in cells stably transfected with vector-based shRNA expression system. *Virus Res.* 135 292–297. 10.1016/j.virusres.2008.04.014 18514349

[B108] Paranjape S. M. Harris E. (2010). Control of dengue virus translation and replication. *Curr. Top. Microbiol. Immunol.* 338 15–34. 10.1007/978-3-642-02215-9_2 19802575

[B109] Pijlman G. P. Funk A. Kondratieva N. Leung J. Torres S. Van Der Aa L. (2008). A highly structured, nuclease-resistant, noncoding RNA produced by flaviviruses is required for pathogenicity. *Cell Host Microbe* 4 579–591. 10.1016/j.chom.2008.10.007 19064258

[B110] Polacek C. Foley J. E. Harris E. (2009a). Conformational changes in the solution structure of the dengue virus 5′ end in the presence and absence of the 3′ untranslated region. *J. Virol.* 83 1161–1166. 10.1128/JVI.01362-08 19004957 PMC2612390

[B111] Polacek C. Friebe P. Harris E. (2009b). Poly(A)-binding protein binds to the non-polyadenylated 3′ untranslated region of dengue virus and modulates translation efficiency. *J. Gen. Virol.* 90 687–692. 10.1099/vir.0.007021-0 19218215

[B112] Proutski V. Gritsun T. S. Gould E. A. Holmes E. C. (1999). Biological consequences of deletions within the 3′-untranslated region of flaviviruses may be due to rearrangements of RNA secondary structure. *Virus Res.* 64 107–123. 10.1016/S0168-1702(99)00079-9 10518708

[B113] Pybus O. G. Rambaut A. Holmes E. C. Harvey P. H. (2002). New inferences from tree shape: numbers of missing taxa and population growth rates. *Syst. Biol.* 51 881–888. 10.1080/10635150290102582 12554454

[B114] Raviprakash K. Liu K. Matteucci M. Wagner R. Riffenburgh R. Carl M. (1995). Inhibition of dengue virus by novel, modified antisense oligonucleotides. *J. Virol.* 69 69–74. 7983769 10.1128/jvi.69.1.69-74.1995 PMC188549

[B115] Ray D. Shah A. Tilgner M. Guo Y. Zhao Y. Dong H. (2006). West Nile virus 5′-cap structure is formed by sequential guanine N-7 and ribose 2’-O methylations by nonstructural protein 5. *J. Virol.* 80 8362–8370. 10.1128/JVI.00814-06 16912287 PMC1563844

[B116] Rice C. M. Lenches E. M. Eddy S. R. Shin S. J. Sheets R. L. Strauss J. H. (1985). Nucleotide sequence of yellow fever virus: implications for flavivirus gene expression and evolution. *Science* 229 726–733. 10.1126/science.4023707 4023707

[B117] Roby J. A. Pijlman G. P. Wilusz J. Khromykh A. A. (2014). Noncoding subgenomic flavivirus RNA: multiple functions in West Nile virus pathogenesis and modulation of host responses. *Viruses* 6 404–427. 10.3390/v6020404 24473339 PMC3939463

[B118] Romero-López C. Berzal-Herranz A. (2013). Unmasking the information encoded as structural motifs of viral RNA genomes: a potential antiviral target. *Rev. Med. Virol.* 23 340–354. 10.1002/rmv.1756 23983005 PMC7169113

[B119] Roth H. Magg V. Uch F. Mutz P. Klein P. Haneke K. (2017). Flavivirus infection uncouples translation suppression from cellular stress responses. *MBio* 8 e02150-16. 10.1128/mBio.02150-16 PMC5225315 28074025

[B120] Rouha H. Hoenninger V. M. Thurner C. Mandl C. W. (2011). Mutational analysis of three predicted 5′-proximal stem-loop structures in the genome of tick-borne encephalitis virus indicates different roles in RNA replication and translation. *Virology* 417 79–86. 10.1016/j.virol.2011.05.008 21645915 PMC3182534

[B121] Saeedi B. J. Geiss B. J. (2013). Regulation of flavivirus RNA synthesis and capping. *Wiley Interdiscip. Rev. RNA* 4 723–735. 10.1002/wrna.1191 23929625 PMC3797245

[B122] Sánchez-Luque F. J. Stich M. Manrubia S. Briones C. Berzal-Herranz A. (2014). Efficient HIV-1 inhibition by a 16 nt-long RNA aptamer designed by combining in vitro selection and in silico optimisation strategies. *Sci. Rep.* 4:6242 10.1038/srep06242 PMC4150108 25175101

[B123] Schnettler E. Sterken M. G. Leung J. Y. Metz S. W. Geertsema C. Goldbach R. W. (2012). Noncoding flavivirus RNA displays RNA interference suppressor activity in insect and mammalian cells. *J. Virol.* 86 13486–13500. 10.1128/JVI.01104-12 23035235 PMC3503047

[B124] Schrauf S. Mandl C. W. Bell-Sakyi L. Skern T. (2009). Extension of flavivirus protein C differentially affects early RNA synthesis and growth in mammalian and arthropod host cells. *J. Virol.* 83 11201–11210. 10.1128/JVI.01025-09 19692461 PMC2772764

[B125] Schuessler A. Funk A. Lazear H. M. Cooper D. A. Torres S. Daffis S. (2012). West Nile virus noncoding subgenomic RNA contributes to viral evasion of the type I interferon-mediated antiviral response. *J. Virol.* 86 5708–5718. 10.1128/JVI.00207-12 22379089 PMC3347305

[B126] Schuster P. (1993). RNA based evolutionary optimization. *Orig. Life Evol. Biosph.* 23 373–391. 10.1007/BF01582087 7509478

[B127] Shi P. Y. Brinton M. A. Veal J. M. Zhong Y. Y. Wilson W. D. (1996). Evidence for the existence of a pseudoknot structure at the 3′ terminus of the flavivirus genomic RNA. *Biochemistry* 35 4222–4230. 10.1021/bi952398v 8672458

[B128] Shurtleff A. C. Beasley D. W. Chen J. J. Ni H. Suderman M. T. Wang H. (2001). Genetic variation in the 3′ non-coding region of dengue viruses. *Virology* 281 75–87. 10.1006/viro.2000.0748 11222098

[B129] Silva P. A. Pereira C. F. Dalebout T. J. Spaan W. J. Bredenbeek P. J. (2010). An RNA pseudoknot is required for production of yellow fever virus subgenomic RNA by the host nuclease XRN1. *J. Virol.* 84 11395–11406. 10.1128/JVI.01047-10 20739539 PMC2953177

[B130] Silva R. L. De Silva A. M. Harris E. MacDonald G. H. (2008). Genetic analysis of Dengue 3 virus subtype III 5′ and 3′ non-coding regions. *Virus Res.* 135 320–325. 10.1016/j.virusres.2008.03.007 18436323

[B131] Song B. H. Yun S. I. Choi Y. J. Kim J. M. Lee C. H. Lee Y. M. (2008). A complex RNA motif defined by three discontinuous 5-nucleotide-long strands is essential for flavivirus RNA replication. *RNA* 14 1791–1813. 10.1261/rna.993608 18669441 PMC2525960

[B132] Suzuki R. Fayzulin R. Frolov I. Mason P. W. (2008). Identification of mutated cyclization sequences that permit efficient replication of West Nile virus genomes: use in safer propagation of a novel vaccine candidate. *J. Virol.* 82 6942–6951. 10.1128/JVI.00662-08 18480453 PMC2446964

[B133] Sztuba-Solinska J. Teramoto T. Rausch J. W. Shapiro B. A. Padmanabhan R. Le Grice S. F. (2013). Structural complexity of dengue virus untranslated regions: *cis*-acting RNA motifs and pseudoknot interactions modulating functionality of the viral genome. *Nucleic Acids Res.* 41 5075–5089. 10.1093/nar/gkt203 23531545 PMC3643606

[B134] Ta M. Vrati S. (2000). Mov34 protein from mouse brain interacts with the 3′ noncoding region of Japanese encephalitis virus. *J. Virol.* 74 5108–5115. 10.1128/JVI.74.11.5108-5115.2000 10799585 PMC110863

[B135] Thurner C. Witwer C. Hofacker I. L. Stadler P. F. (2004). Conserved RNA secondary structures in Flaviviridae genomes. *J. Gen. Virol.* 85 1113–1124. 10.1099/vir.0.19462-0 15105528

[B136] Tilgner M. Deas T. S. Shi P. Y. (2005). The flavivirus-conserved penta-nucleotide in the 3′ stem-loop of the West Nile virus genome requires a specific sequence and structure for RNA synthesis, but not for viral translation. *Virology* 331 375–386. 10.1016/j.virol.2004.07.022 15629780

[B137] Tsetsarkin K. A. Liu G. Shen K. Pletnev A. G. (2016). Kissing-loop interaction between 5′ and 3′ ends of tick-borne Langat virus genome ‘bridges the gap’ between mosquito- and tick-borne flaviviruses in mechanisms of viral RNA cyclization: applications for virus attenuation and vaccine development. *Nucleic Acids Res.* 44 3330–3350. 10.1093/nar/gkw061 26850640 PMC4838367

[B138] Tuplin A. Evans D. J. Buckley A. Jones I. M. Gould E. A. Gritsun T. S. (2011). Replication enhancer elements within the open reading frame of tick-borne encephalitis virus and their evolution within the Flavivirus genus. *Nucleic Acids Res.* 39 7034–7048. 10.1093/nar/gkr237 21622960 PMC3303483

[B139] Urosevic N. Van Maanen M. Mansfield J. P. Mackenzie J. S. Shellam G. R. (1997). Molecular characterization of virus-specific RNA produced in the brains of flavivirus-susceptible and -resistant mice after challenge with Murray Valley encephalitis virus. *J. Gen. Virol.* 78(Pt 1), 23–29. 10.1099/0022-1317-78-1-23 9010281

[B140] Vashist S. Anantpadma M. Sharma H. Vrati S. (2009). La protein binds the predicted loop structures in the 3′ non-coding region of Japanese encephalitis virus genome: role in virus replication. *J. Gen. Virol.* 90 1343–1352. 10.1099/vir.0.010850-0 19264640

[B141] Villordo S. M. Alvarez D. E. Gamarnik A. V. (2010). A balance between circular and linear forms of the dengue virus genome is crucial for viral replication. *RNA* 16 2325–2335. 10.1261/rna.2120410 20980673 PMC2995394

[B142] Villordo S. M. Carballeda J. M. Filomatori C. V. Gamarnik A. V. (2016). RNA Structure duplications and flavivirus host adaptation. *Trends Microbiol.* 24 270–283. 10.1016/j.tim.2016.01.002 26850219 PMC4808370

[B143] Villordo S. M. Gamarnik A. V. (2013). Differential RNA sequence requirement for dengue virus replication in mosquito and mammalian cells. *J. Virol.* 87 9365–9372. 10.1128/JVI.00567-13 23760236 PMC3754043

[B144] Wang E. Weaver S. C. Shope R. E. Tesh R. B. Watts D. M. Barrett A. D. (1996). Genetic variation in yellow fever virus: duplication in the 3′ noncoding region of strains from Africa. *Virology* 225 274–281. 10.1006/viro.1996.0601 8918913

[B145] Ward A. M. Bidet K. Yinglin A. Ler S. G. Hogue K. Blackstock W. (2011). Quantitative mass spectrometry of DENV-2 RNA-interacting proteins reveals that the DEAD-box RNA helicase DDX6 binds the DB1 and DB2 3′ UTR structures. *RNA Biol.* 8 1173–1186. 10.4161/rna.8.6.17836 21957497 PMC3256426

[B146] Wei Y. Qin C. Jiang T. Li X. Zhao H. Liu Z. (2009). Translational regulation by the 3′ untranslated region of the dengue type 2 virus genome. *Am. J. Trop. Med. Hyg.* 81 817–824. 10.4269/ajtmh.2009.08-0595 19861617

[B147] Welsch S. Miller S. Romero-Brey I. Merz A. Bleck C. K. Walther P. (2009). Composition and three-dimensional architecture of the dengue virus replication and assembly sites. *Cell Host Microbe* 5 365–375. 10.1016/j.chom.2009.03.007 19380115 PMC7103389

[B148] Wengler G. (1981). Terminal sequences of the genome and replicative-from RNA of the flavivirus West Nile virus: absence of poly(A) and possible role in RNA replication. *Virology* 113 544–555. 10.1016/0042-6822(81)90182-3 7269253

[B149] Wengler G. Castle E. (1986). Analysis of structural properties which possibly are characteristic for the 3′-terminal sequence of the genome RNA of flaviviruses. *J. Gen. Virol.* 67(Pt 6), 1183–1188. 10.1099/0022-1317-67-6-1183 3011975

[B150] Wengler G. Castle E. Leidner U. Nowak T. (1985). Sequence analysis of the membrane protein V3 of the flavivirus West Nile virus and of its gene. *Virology* 147 264–274. 10.1016/0042-6822(85)90129-1 3855247

[B151] Yang Y. Wu C. Wu J. Nerurkar V. R. Yanagihara R. Lu Y. (2008). Inhibition of West Nile Virus replication by retrovirus-delivered small interfering RNA in human neuroblastoma cells. *J. Med. Virol.* 80 930–936. 10.1002/jmv.21164 18360908 PMC2825143

[B152] You S. Falgout B. Markoff L. Padmanabhan R. (2001). In vitro RNA synthesis from exogenous dengue viral RNA templates requires long range interactions between 5′- and 3′-terminal regions that influence RNA structure. *J. Biol. Chem.* 276 15581–15591. 10.1074/jbc.M010923200 11278787

[B153] Yu L. Markoff L. (2005). The topology of bulges in the long stem of the flavivirus 3′ stem-loop is a major determinant of RNA replication competence. *J. Virol.* 79 2309–2324. 10.1128/JVI.79.4.2309-2324.2005 15681432 PMC546603

[B154] Yu L. Nomaguchi M. Padmanabhan R. Markoff L. (2008). Specific requirements for elements of the 5′ and 3′ terminal regions in flavivirus RNA synthesis and viral replication. *Virology* 374 170–185. 10.1016/j.virol.2007.12.035 18234265 PMC3368002

[B155] Zeng L. Falgout B. Markoff L. (1998). Identification of specific nucleotide sequences within the conserved 3′-SL in the dengue type 2 virus genome required for replication. *J. Virol.* 72 7510–7522. 9696848 10.1128/jvi.72.9.7510-7522.1998 PMC109990

[B156] Zhang B. Dong H. Stein D. A. Iversen P. L. Shi P. Y. (2008a). West Nile virus genome cyclization and RNA replication require two pairs of long-distance RNA interactions. *Virology* 373 1–13. 10.1016/j.virol.2008.01.016 18258275

[B157] Zhang B. Dong H. Stein D. A. Shi P. Y. (2008b). Co-selection of West Nile virus nucleotides that confer resistance to an antisense oligomer while maintaining long-distance RNA/RNA base pairings. *Virology* 382 98–106. 10.1016/j.virol.2008.08.044 18842280 PMC3202013

[B158] Zhang B. Dong H. Zhou Y. Shi P. Y. (2008c). Genetic interactions among the West Nile virus methyltransferase, the RNA-dependent RNA polymerase, and the 5′ stem-loop of genomic RNA. *J. Virol.* 82 7047–7058. 10.1128/JVI.00654-08 18448528 PMC2446981

[B159] Zhou Y. Ray D. Zhao Y. Dong H. Ren S. Li Z. (2007). Structure and function of flavivirus NS5 methyltransferase. *J. Virol.* 81 3891–3903. 10.1128/JVI.02704-06 17267492 PMC1866096

